# Development and Validation of a Risk Model to Predict Intraoperative Blood Transfusion

**DOI:** 10.1001/jamanetworkopen.2025.5522

**Published:** 2025-04-17

**Authors:** Annika Eyth, Felix Borngaesser, Maíra I. Rudolph, Béla-Simon Paschold, Tina Ramishvili, Lars Kaiser, Christopher W. Tam, Karuna Wongtangman, Greta Eikermann, Shweta Garg, Michael H. Karasick, Michael E. Kiyatkin, Milan M. Kinkhabwala, Stephen J. Forest, Jonathan Leff, Ling Zhang, Philipp Fassbender, Ibraheem Karaye, Andrea U. Steinbicker, Maximilian S. Schaefer, Matthias Eikermann, Se-Chan Kim

**Affiliations:** 1 Department of Anesthesiology, Montefiore Medical Center, Albert Einstein College of Medicine, Bronx, New York; 2 University Clinic for Anesthesiology, Intensive Care, Emergency Medicine, and Pain Therapy, Carl von Ossietzky Universität Oldenburg and Klinikum Oldenburg AöR, Oldenburg, Germany; 3 University of Cologne, Faculty of Medicine and University Hospital Cologne, Department of Anesthesiology and Intensive Care Medicine, Cologne, Germany; 4 Department of Anesthesia, Critical Care and Pain Medicine, Beth Israel Deaconess Medical Center, Harvard Medical School, Boston, Massachusetts; 5 Center for Anesthesia Research Excellence (CARE), Beth Israel Deaconess Medical Center, Boston, Massachusetts; 6 Department of Anesthesiology, Faculty of Medicine, Siriraj Hospital, Mahidol University, Bangkok, Thailand; 7 Ethical Culture Fieldston School, Bronx, New York; 8 Montefiore Einstein Center for Health Data Innovations, Montefiore Medical Center, Albert Einstein College of Medicine, Bronx, New York; 9 Department of Pathology, Montefiore Medical Center, Albert Einstein College of Medicine, Bronx, New York; 10 Department of Transplant and Hepatobiliary Surgery, Montefiore Medical Center, Albert Einstein College of Medicine, Bronx, New York; 11 Cardiovascular And Thoracic Surgery, Montefiore Medical Center, Albert Einstein College of Medicine, Bronx, New York; 12 Department of Anesthesiology, University Hospital Duesseldorf, Duesseldorf, Germany; 13 Klinik für Anästhesiologie und Intensivmedizin, Universität Duisburg-Essen, Essen, Germany; 14 Department of Anesthesiology and Intensive Care Medicine, University Hospital Bonn, Bonn, Germany

## Abstract

**Importance:**

Crossmatched packed red blood cells (pRBC) that are not transfused result in significant waste of this scarce resource. Efficient utilization should be part of a patient blood management strategy.

**Objective:**

To develop and validate a prediction model to identify surgical patients at high risk of intraoperative pRBC transfusion.

**Design, Setting, and Participants:**

This prognostic study used hospital registry data from 2 quaternary hospital networks from January 2016 to June 2021 (development: Montefiore Medical Center [MMC], Bronx, New York), June 2021 to February 2023 (internal validation: MMC), and January 2008 to June 2022 (external validation: Beth Israel Deaconess Medical Center [BIDMC], Boston, Massachusetts). Participants were patients aged 18 years or older undergoing surgery.

**Main Outcome and Measures:**

The outcome was intraoperative transfusion of 1 or more pRBC units. Based on a priori–defined candidate predictors, stepwise backward regression was applied to develop a computational model of independent predictors for intraoperative pRBC transfusion.

**Results:**

The development and validation cohorts consisted of 816 618 patients (273 654 at MMC: mean [SD], age 57.5 [17.2] years; 161 481 [59.0%] female; 542 964 at BIDMC: mean [SD] age, 56.0 [17.1] years; 310 272 [57.1%] female). Overall, 18 662 patients (2.3%) received at least 1 unit of pRBC. The final model contained 24 preoperative predictors: nonambulatory surgery; American Society of Anesthesiologists physical status; international normalized ratio; redo surgery; emergency surgery or surgery outside of regular working hours; estimated surgical duration of at least 120 minutes; surgical complexity; liver disease; hypoalbuminemia; thrombocytopenia; mild, moderate, or severe anemia; and surgery type. The area under the receiver operating characteristic curve (AUC) was 0.93 (95% CI, 0.92-0.93), suggesting high predictive accuracy and generalizability. Positive predictive value (PPV) and negative predictive value (NPV) were 8.9% (95% CI, 8.7%-9.2%) and 99.7% (95% CI, 99.7%-99.7%), respectively, with increased predictive values for operations with a higher a priori risk of pRBC transfusion. The model’s performance was confirmed in internal and external validation. The prediction tool outperformed the established Transfusion Risk Understanding Scoring Tool (AUC, 0.64 [0.63-0.64]; PPV, 2.6% [95% CI, 2.5%-2.6%]; NPV, 99.2% [95% CI, 99.1%-99.3%]) (*P* < .001) and was noninferior to 3 machine learning–derived scores.

**Conclusions and Relevance:**

In this prognostic study of surgical patients, the Transfusion Forecast Utility for Surgical Events (TRANSFUSE) model for predicting intraoperative pRBC transfusion was developed and validated. The instrument can be used independently of machine learning infrastructure availability to inform preoperative pRBC orders and to minimize waste of nontransfused red blood cell units.

## Key Points

**Question:**

Can the need for intraoperative packed red blood cell transfusions be reliably predicted using an easily implemented instrument?

**Findings:**

In this prognostic study with 816 618 patients undergoing surgery in 2 different academic centers, 2.3% received at least 1 unit of packed red blood cells during surgery. The final model consisted of 24 preoperative predictors, and its predictive accuracy was comparable with machine learning models.

**Meaning:**

The model developed and validated in this study can be used to predict intraoperative packed red blood cell transfusion utilization, which will help clinicians optimize preoperative blood orders and reduce waste of red blood cell units.

## Teaser

This prognostic study develops and validates a new model for projecting the amount of packed red blood cells needed during surgery.

## Introduction

For the first time in its history, the American Red Cross declared a National Blood Emergency in January 2022.^1,2^ Surgical services account for a substantial amount of the blood usage in hospitals, and it is unclear how to allocate blood supply to various types and complexities of surgery.^3,4,5^ One unit of packed red blood cells (pRBC) costs up to approximately $1183 for the complete process of pRBC transfusion in the United States, €435 at average in Europe, and £158 for the purchase of a pRBC unit in the United Kingdom.^6,7,8^ Blood issued but not transfused is not only an economic health care burden, but also contributes to the global shortage.^8^ Consequently, there is a demand for accurate prediction of intraoperative pRBC transfusion requirements. Existing prediction models for intraoperative pRBC transfusion were developed for specific surgical services, such as cardiac, orthopedic, or liver transplant surgical procedures, yet lack generalizability.^4,5,9^ Although machine learning prediction tools are recognized more and more,^10^ they may not be easy to implement across institutions with variable information technology resources. We aimed to create an instrument to predict intraoperative pRBC transfusion across a wide range of surgical procedures that is generalizable and can be implemented and used to inform clinical practice in a wide range of hospital settings.

## Methods

### Study Cohort and Data Collection

All adult patients (≥18 years) undergoing surgery at the 2 study sites were eligible for inclusion. Patients with an American Society of Anesthesiologists (ASA) physical status score of 6 were excluded from the study.

For score development, we included all patients who underwent surgery at Montefiore Medical Center (MMC), Bronx, New York, between January 2016 and June 2021. For internal model validation, we analyzed MMC data from June 2021 to February 2023. Data from Beth Israel Deaconess Medical Center (BIDMC), Boston, Massachusetts, between January 2008 and June 2022 were used for external model validation (eMethods 1 in Supplement 1).

Data were retrieved from electronic hospital management databases between June 2023 and November 2024. Both institutional review boards approved this study with a waiver of informed consent because all included patient data were collected in routine clinical operations. Data were handled retrospectively and strictly deidentified, which excludes disadvantages for patients. Our study complies with the Health Insurance Portability and Accountability Act requirements for confidentiality. This article adheres to both the Transparent Reporting of a Multivariable Prediction Model for Individual Prognosis or Diagnosis (TRIPOD)^11^ and Consolidated Health Economic Evaluation Reporting Standards (CHEERS)^12^ reporting guidelines.

Missing data were analyzed and subsequently imputed. Detailed information on the applied methods is provided in the eMethods 2, eFigure 1, and eFigure 2 in Supplement 1.

Race and ethnicity data were sourced from the electronic health record, based on self-reported intake questionnaires completed at hospital admission or during prior visits. At MMC, the ethnicity questionnaire offered 3 response options: “Spanish/Hispanic/Latino,” “Not Spanish/Hispanic/Latino,” and “Declined,” in alignment with US Census Bureau guidelines.^13^ The race questionnaire included the categories “American Indian or Alaska Native,” “Asian,” “Black or African American,” “Native Hawaiian or Other Pacific Islander,” “Mixed Race,” “White,” “Other,” and “Declined/Unavailable.” Following the classification framework of the Centers for Disease Control and Prevention,^14^ patients were assigned to mutually exclusive racial and ethnic groups. Those who chose not to disclose their race and ethnicity or whose information was unavailable were categorized as “Declined/Unknown.” Race and ethnicity data were collected to characterize the cohort and provide context for social factors that may influence the risk of blood transfusion (eTable 12 in Supplement 1). Additionally, because the racial and ethnic composition of the development and external validation cohorts differed substantially, this supports the applicability of our findings to diverse populations.

### Intraoperative pRBC Transfusion and Potential Predictors

The outcome was the intraoperative transfusion of at least 1 unit of pRBC. The application of an autologous blood recovery system was not included as an outcome.

Potential predictors of pRBC transfusion were selected based on comprehensive literature review and clinical plausibility. These included baseline demographic characteristics, preoperative comorbidities, surgery types, and surgical complexity and duration (eTables 1 and 2 in Supplement 1).^15,16,17^ To further explore the differences in pRBC transfusion in patients with preoperative no, mild, moderate, or severe anemia, we investigated the use of vasopressors measured as cumulative norepinephrine equivalent dose in these patient groups (eMethods 3 in Supplement 1).

### Score Development and Validation

We used stepwise backward regression to identify outcome predictors, additionally accounting for overfitting. The final item score values were based on the respective β coefficients (eMethods 4 in Supplement 1). The Transfusion Forecast Utility for Surgical Events (TRANSFUSE) score’s performance was assessed using *C* statistics. Cutoffs for low and high risk of transfusion were determined using the Youden Index. Detailed information and additionally applied measures of precision are outlined in eMethods 5 and eFigure 3 in Supplement 1. The score was internally and externally validated in independent cohorts.

### Sensitivity and Exploratory Analysis

We compared the discriminatory ability of our prediction model with the previously published Transfusion Risk Understanding Scoring Tool (TRUST) score within our developmental and external validation cohort^9^ and with 3 machine learning–derived models (eMethods 6 in Supplement 1). The performance of the score was evaluated across patient subgroups undergoing different surgery types, such as cardiac, transplant, cancer, gastrointestinal, or musculoskeletal surgery, and other surgical procedures of known high risk for pRBC transfusion (eTable 3 in Supplement 1).^18^ A detailed definition and baseline characteristics of all subgroups can be found in the online supplement (eTables 4-9 in Supplement 1). We further assessed the score’s performance in predicting massive pRBC transfusion, defined as the intraoperative administration of either more than 3 or 10 pRBC units.^19,20^

To confirm the robustness of the score, we also investigated its predictive value for intraoperative transfusion of fresh frozen plasma (FFP), defined as at least 1 intraoperative transfusion of FFP. Finally, we assessed the score’s performance with an included additional predictor: perioperative administration of tranexamic acid.

### Statistical Analysis

Continuous, normally distributed variables are expressed as means with SDs or standard errors of the mean (SEMs). Skewed variables are presented as medians with IQRs, and categorical variables as frequency counts with percentages. For the score development, we used stepwise backward logistic regression. Group differences were analyzed using Student *t* test, and a 2-sided *P* < .05 was considered statistically significant (eMethods 3 in Supplement 1). Data were analyzed using Stata software version 17 (StataCorp) and Prism 10 (GraphPad).

## Results

### Study Design and Population

Overall, 816 618 patients (273 654 at MMC: mean [SD], age 57.5 [17.2] years; 161 481 [59.0%] female; 80 349 [29.4%] Black, 106 978 [39.1%] Hispanic, and 38 570 [14.1%] White patients; 542 964 at BIDMC: mean [SD] age, 56.0 [17.1] years; 310 272 [57.1%] female; 54 054 [10.0%] Black, 28 359 [5.2%] Hispanic, and 373 663 [68.6%] White patients) were included in the study. The final developmental study cohort at MMC consisted of 205 200 adult patients (mean [SD] age, 57.0 [17.2] years, 81 877 [60.1%] female and 123 323 [39.9%] male patients) undergoing surgical procedures after applying exclusion criteria. Of these patients, 4031 (2.0%) received at least 1 intraoperative transfusion of pRBC.

**Figure 1.**
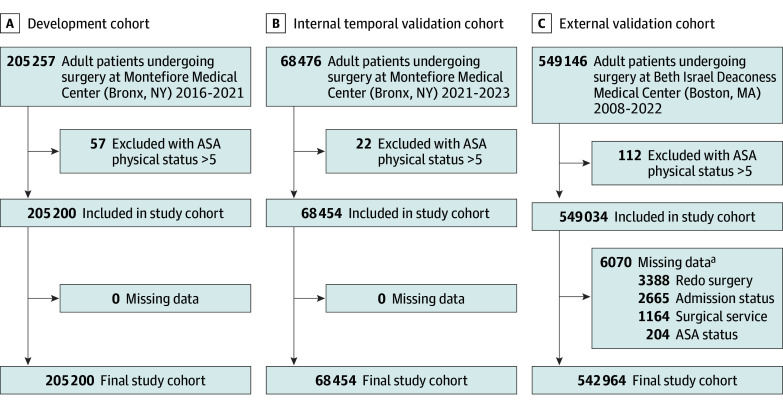
Study Flow Data from Montefiore Medical Center in the Bronx, New York, were divided chronologically into the development cohort (January 2016 to June 2021) (A) and internal validation cohort (June 2021 to February 2023) (B). C, Data from Beth Israel Deaconess Medical Center in Boston, Massachusetts, were used as an external validation cohort (January 2008 to June 2022). ASA indicates American Society of Anesthesiologists. ^a^Multiple criteria may apply.

The cohort used for prospective validation comprised 68 454 patients, who underwent surgery at MMC between June 2021 and February 2023 (mean [SD] age, 58.9 [16.7] years; 30 296 [55.7%] female and 38 158 [44.3%] male), with 1468 (2.1%) requiring intraoperative pRBC transfusions. The final validation cohort at BIDMC included 542 964 adult surgical patients after applying exclusion criteria, among whom 13 163 (2.4%) received at least 1 unit of pRBC (Figure 1 and Table 1; eTable 10 in Supplement 1) At MMC, 122 525 of 273 654 patients (44.8%) presented with anemia of any severity preoperatively; of those, 36 148 (29.5%) underwent nonelective surgery.

**Table 1.** Baseline Characteristics of Development, Internal, and External Validation Cohorts

Characteristics	Patients, No. (%)			
	Montefiore Medical Center		Beth Israel Deaconess Medical Center	
	No intraoperative transfusion (n = 268 155)	Intraoperative transfusion (n = 5499)	No intraoperative transfusion (n = 529 801)	Intraoperative transfusion (n = 13 163)
Age, mean (SD), y	57.4 (17.1)	59.3 (17.0)	55.8 (17.1)	63.5 (16.2)
Sex assigned at birth				
Female	158 488 (59.1)	2993 (54.4)	303 765 (57.3)	6507 (49.4)
Male	109 667 (40.9)	2506 (45.6)	225 983 (42.7)	6656 (50.6)
Race/ethnicity				
Asian	7049 (2.6)	199 (3.6)	25 997 (4.9)	457 (3.5)
Black	78 586 (29.3)	1763 (32.1)	52 885 (10.0)	1169 (8.9)
Hispanic	105 185 (39.2)	1793 (32.6)	27 798 (5.2)	561 (4.3)
White	37 638 (14.0)	932 (17.0)	364 243 (68.8)	9420 (71.6)
Additional groups^a^	18 756 (7.0)	443 (8.1)	53 374 (10.1)	1445 (11.0)
Unavailable or declined	20 855 (7.8)	366 (6.7)	5495 (1.0)	110 (0.8)
Nonambulatory surgery	155 264 (57.9)	5453 (99.2)	230 880 (43.6)	12 855 (97.7)
High ASA Status, >2	164 537 (61.4)	4725 (85.9)	232 722 (45.2)	11 441 (86.9)
Emergency or started outside of regular working hours	33 089 (12.3)	2071 (37.7)	37 304 (7.0)	2159 (16.4)
High surgical complexity	118 296 (44.1)	4721 (85.9)	251 027 (47.4)	12 022 (91.3)
Estimated surgical duration ≥120 min	105 064 (39.2)	4758 (86.5)	181 133 (34.2)	11 374 (86.4)
Mild anemia	75 397 (28.1)	1549 (28.2)	95 617 (17.9)	6980 (53.0)
Moderate anemia	36 585 (13.6)	2490 (45.3)	1021 (0.2)	110 (0.8)
Severe anemia	5614 (2.1)	890 (16.2)	113 (0.0)	33 (0.3)
Liver disease, bilirubin ≥2 mg/dL	38 857 (14.5)	1524 (27.7)	43 035 (8.1)	2122 (16.1)
Kidney failure, GFR <15 mL/min/1.73 m^2^	51 228 (19.1)	2134 (38.8)	40 213 (7.6)	2879 (21.9)
Hypoalbuminemia	21 320 (8.0)	1449 (26.4)	771 (0.1)	79 (0.6)
Thrombocytopenia	15 406 (5.7)	887 (16.1)	719 (0.1)	70 (0.5)
Abnormal preoperative INR ratio, >1.2 and <2.0	17 087 (6.4)	1274 (23.2)	733 (0.1)	71 (0.5)
Redo surgery	3319 (1.2)	282 (5.1)	4752 (0.9)	428 (3.3)
Surgery on cardiopulmonary bypass	3059 (1.1)	833 (15.1)	8854 (1.7)	3236 (24.6)
Obstetric/gynecological surgery	17 804 (6.6)	563 (10.2)	93 031 (17.6)	912 (6.9)
Urological surgery	8240 (3.1)	159 (2.9)	23 050 (4.4)	273 (2.1)
Cardiac surgery, off pump	2919 (1.1)	224 (4.1)	11 569 (2.2)	158 (1.2)
Thoracic surgery, off pump	5362 (2.0)	175 (3.2)	40 587 (7.7)	366 (2.8)
Vascular surgery	17 279 (6.4)	879 (16.0)	15 069 (2.8)	1649 (12.5)
Orthopedic surgery	32 814 (12.2)	994 (18.1)	91 689 (17.3)	3439 (26.1)
Neurosurgery	5996 (2.2)	259 (4.7)	17 533 (3.3)	508 (3.9)
Major abdominal surgery	32 833 (12.2)	828 (15.1)	83 635 (15.8)	1200 (9.1)
Visceral transplant surgery	1124 (0.4)	279 (5.1)	1739 (0.3)	611 (4.6)
Plastic surgery	3072 (1.1)	69 (1.3)	21 443 (23.0)	182 (1.4)

Abbreviations: ASA, American Society of Anesthesiologists; GFR, glomerular filtration rate; INR, international normalized ratio.

SI conversion factor: To convert bilirubin to micromoles per liter, multiply by 17.104.

^a^ Additional racial and ethnic groups include American Indian, Alaska Native, Native Hawaiian or Pacific Islander, and mixed race (non-Hispanic) individuals.

Upon analysis of the data from the combined 3 cohorts, among the 18 662 patients (2.3%) who received an intraoperative pRBC transfusion, 6610 (35.4%) did not have anemia, 8529 (45.7%) had mild preoperative anemia (hemoglobin [Hb] <12 g/dL for female patients and <13 g/dL for male patients [to convert to grams per liter, multiply by 10]), 2600 (13.9%) had moderate anemia (Hb <10 g/dL to >7.5 g/dL), and 923 (4.9%) had severe anemia (Hb <7.5 g/dL). Among patients who received vs did not receive red blood cell transfusions, 3129 (77.6%) vs 54 622 (27.2%) were treated with intraoperative vasopressors. Patients with only mild anemia treated with red blood cell transfusions were often hemodynamically unstable; they received a significantly higher mean (SD) cumulative dose of norepinephrine equivalent (1.04 [0.17] mg; 1592 patients) compared with those with moderate or severe anemia (0.66 [0.06] mg; 2439 patients) (*P* = .007) (eFigure 4 in Supplement 1).

### Score Development

Following stepwise backward regression, a total of 24 predictors from 30 candidate predictors were retained in our final prediction model, including surgery type, baseline demographics, preoperative data, and comorbidities. The final predictors included in the computational model were nonambulatory surgery; an ASA physical status greater than 2; abnormal international normalized ratio; redo surgery; emergency surgery or surgery performed outside of regular working hours; estimated surgical duration of 120 minutes or longer; high surgical complexity; liver disease; hypoalbuminemia (albumin <3.5 g/dL [to convert to grams per liter, multiply by 10]); thrombocytopenia (platelets <150 × 10^3^/mL [to convert to ×10^9^ per liter, multiply by 1]); mild, moderate, and severe anemia; and abdominal, plastic, vascular, thoracic off-pump, urological, orthopedic or trauma, cardiac on-pump and off-pump, obstetric/gynecological, and visceral transplant surgery and neurosurgery.

**Figure 2.**
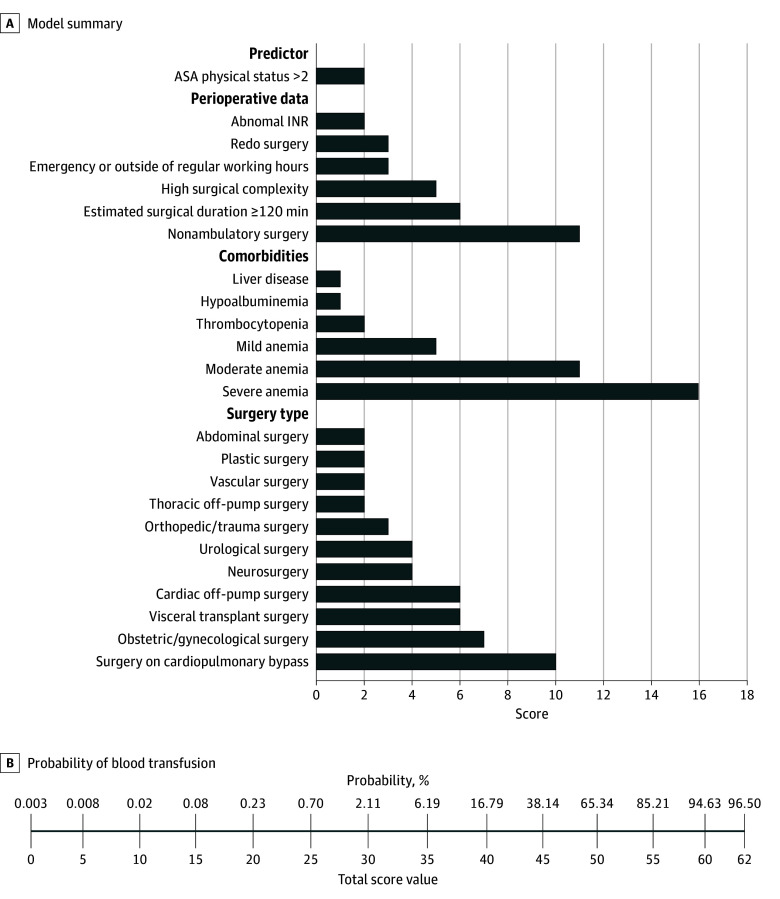
Summary Figure A, The variables included in the model are summarized with corresponding score point values. The exact definitions of each predictor can be found in eTables 3 and 4 in Supplement 1. B, Probability of intraoperative packed red blood cell transfusion as percentages (0.003%-96.5%) in reference to the total score value that a patient can receive (0-62). ASA indicates American Society of Anesthesiologists; INR, international normalized ratio.

The assigned weighting values ranged from 1 to 16, with a value of 16 assigned to severe anemia, while the maximum score reached 62. The probabilities of intraoperative pRBC transfusion varied from 0.003% to 96.5%. (Figure 2; eTable 11 in Supplement 1).

### Score Performance

**Figure 3.**
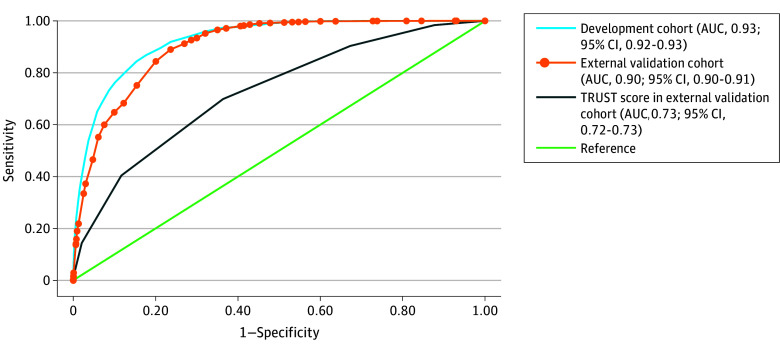
Model Discrimination AUC indicates area under the receiver operating characteristic curve; TRUST, Transfusion Risk Understanding Scoring Tool.

In the training cohort, the score achieved an area under the receiver operating characteristic curve (AUC) of 0.93 (95% CI, 0.92-0.93) indicating excellent discriminative ability. The Brier score was 0.02 (Figure 3).

The Youden *J* statistic identified a cut point of 30 or greater. Consequently, 166 067 patients (80.9%) were classified as low risk and 39 133 (19.1%) as high risk for intraoperative pRBC transfusion, with sensitivity of 86.8% (95% CI, 85.8%-87.8%) and specificity of 82.3% (95% CI, 82.1%-82.5%). Most intraoperative pRBC transfusions occurred in the high-risk group (3499 of 4031 transfusions [86.8%]). At this cut point, the positive predictive value (PPV) was 8.9% (95% CI, 8.7%-9.2%), and the negative predictive value (NPV) was 99.7% (95% CI, 99.7%-99.7%).

### Internal Validation

The model predicted intraoperative pRBC transfusion with an AUC of 0.92 (95% CI, 0.92-0.93) in the internal validation cohort. Using the predefined cut point of 30 or greater to categorize patients into high-risk and low-risk groups, sensitivity in the prospective validation cohort was 89.6% (95% CI, 87.9%-91.1%), while the specificity was 78.7% (95% CI, 78.4%-79.0%) (PPV, 8.4% [95% CI, 8.0%-8.9%]; NPV, 99.7% [95% CI, 99.7%-99.8%]).

### External Validation

In the external validation cohort, the model demonstrated an excellent discriminative ability with an AUC of 0.90 (95% CI, 0.90-0.90), and no recalibration was required (Figure 3). Utilizing the predefined cut point of 30 or greater to define high-risk and low-risk groups, PPV and NPV were 16.5% (95% CI, 16.2%-16.9%) and 98.9% (95% CI, 98.9%-99.0%), respectively, with sensitivity of 60.0% (95% CI, 59.1%-60.8%) and specificity of 92.5% (95% CI, 92.4%-92.5%).

### Comparison With TRUST Score and Machine Learning Tools

When comparing our score with the TRUST score, our instrument demonstrated a significantly higher AUC of 0.93 (95% CI, 0.92-0.93) vs 0.64 (95% CI, 0.63-0.64) (*P* < .001). This finding was reproduced in the external validation cohort, with AUCs of 0.90 (95% CI, 0.90-0.90) vs 0.73 (95% CI, 0.72-0.73) (*P* < .001) (eFigure 5 in Supplement 1). Additionally, the predictive accuracy of our model was comparable with 3 machine learning–derived instruments (eTable 12 in Supplement 1).

### Additional Clinical Application

The model accurately predicted intraoperative pRBC transfusion across various surgical procedures with high risk of intraoperative transfusion, such as pancreatectomy, distinct open-heart surgical procedures, and liver resection (Table 2). Its discriminative ability was further confirmed for multiple surgical specialties, including transplantation, cancer, and musculoskeletal surgery (eTable 3 in Supplement 1).

**Table 2.** Performance of Model to Predict Intraoperative Transfusion in Selected High-Risk Surgical Procedures

Procedure	≥1 Unit			≥3 Units		
	Incidence of transfusion, No./total No. (%)	PPV (95% CI), %	NPV (95% CI), %	Incidence of transfusion, No./total No. (%)	PPV (95% CI), %	NPV (95% CI), %
Cardiac valve replacement	156/957 (16.3)	44.7 (37.5-52.1)	90.7 (88.5-92.7)	28/957 (2.9)	7.9 (4.5-12.7)	98.3 (97.1-99.1)
Coronary artery bypass graft	189/1460 (12.9)	29.6 (25.2-34.1)	93.8 (92.2-95.2)	32/1460 (2.2)	5.7 (3.7-8.3)	99.2 (98.5-99.7)
Aortic aneurysm repair	54/149 (36.2)	60.0 (40.6-77.3)	69.7 (60.7-77.8)	21/149 (14.1)	33.3 (17.3-52.8)	90.8 (84.1-95.3)
Open femoral fracture repair	39/368 (10.6)	22.4 (15.6-30.4)	96.2 (92.8-98.2)	4/368 (1.1)	0.7 (0.0-4.1)	98.7 (96.3-99.7)
Open total nephrectomy	22/66 (33.3)	56.5 (34.5-76.8)	79.1 (64.0-90.0)	10/66 (15.2)	30.4 (13.2-52.9)	93.0 (80.9-98.5)
Abdominal/retroperitoneal tumor excision >10 cm	3/5 (60.0)	75.0 (19.4-99,.4)	100.0 (2.5-100.0)	NA^a^	NA^a^	NA^a^
Vascular bypass	78/467 (16.7)	38.9 (30.3-48.0)	91.5 (88.0-94.2)	19/467 (4.1)	8.7 (4.4-15.1)	97.7 (95.4-99.0)
Splenectomy	4/69 (5.8)	25.0 (5.5-57.2)	98.2 (90.6-100.0)	NA^a^	NA^a^	NA^a^
Amputation of leg	130/906 (14.3)	19.8 (16.1-23.9)	90.6 (87.6-93.0)	6/906 (0.7)	1.2 (0.4-2.7)	99.8 (99.8-100.0)
Pancreatectomy	39/254 (66.7)	52.0 (37.4-66.3)	93.6 (89.3-96.6)	8/254 (3.1)	8.0 (2.2-19.2)	98.0 (95.1-99.5)
Liver resection	25/261 (9.6)	19.1 (12.4-27.5)	97.9 (94.1-99.6)	8/261 (3.1)	6.1 (2.5-12.1)	99.3 (96.2-100.0)
Resection of bowel or rectum	32/456 (7.0)	15.6 (9.4-23.8)	95.7 (93.0-97.6)	2/456 (0.4)	1.8 (0.2-6.5)	100.0 (98.9-100.0)
Spinal arthrodesis	53/576 (9.2)	16.8 (11.8-22.7)	94.7 (92.0-96.7)	9/576 (1.6)	3.6 (1.4-7.2)	99.5 (98.1-99.9)
Arterial embolectomy	10/59 (16.9)	23.5 (10.7-41.2)	92.0 (74.0-99.0)	NA^a^	NA^a^	NA^a^
Gastrectomy	5/112 (4.5)	15.2 (5.1-31.9)	100.0 (95.4-100.0)	1/112 (0.9)	3.0 (0.1-15.8)	100.0 (95.4-100.0)
Myomectomy	22/271 (8.1)	12.7 (7.7-19.3)	96.9 (92.3-99.1)	4/271 (1.5)	2.8 (0.8-7.1)	100.0 (97.2-100.0)
Open radical prostatectomy	3/99 (3.0)	25.0 (3.2 - 65.1)	98.9 (94.0-100.0)	NA^a^	NA^a^	NA^a^
Total abdominal hysterectomy	80/876 (9.1)	18.9 (14.5-23.9)	95.5 (93.5-97.0)	22/876 (2.5)	5.7 (3.3-9.1)	99.0 (97.8-99.6)
Endovascular aortic aneurysm repair	21/130 (16.2)	24.7 (15.6-35.8)	96.2 (87.0-99.5)	3/130 (2.3)	3.9 (0.8-11.0)	100.0 (93.3-100.0)
Liver transplant	140/215 (65.1)	69.9 (62.1-76.5)	51.1 (36.1-65.9)	121/215 (56.3)	61.3 (53.5-68.7)	61.7 (46.4-75.5)

Abbreviation: NA, not applicable.

^a^ No events recorded.

Overall, 835 (0.4%) and 71 (0.03%) patients received more than 3 or more than 10 pRBC units during surgery, respectively. The model showed excellent discriminative ability for those events (≥3 units: AUC, 0.93 [95% CI, 0.93-0.94]; ≥10 units: AUC, 0.97 [95% CI, 0.96-0.99]). This was further assessed in high-risk surgical procedures for 3 or more pRBC units (Table 2).

A total of 1131 patients (0.6%) at MMC received at least 1 unit of FFP during surgery. The model showed excellent prediction of FFP transfusion (AUC, 0.93 [95% CI, 0.93-0.94]; PPV, 2.6% [95% CI, 2.4%-2.7%]; NPV, 99.9% [95% CI, 99.9%-99.9%]).

The created alternative prediction instrument including perioperative administration of tranexamic acid performed similarly to the original model. Detailed results are displayed in eFigures 6 and 7 in Supplement 1.

## Discussion

In a large cohort of surgical patients from 2 academic health care networks in the United States, we developed and validated internally and externally a novel tool to predict intraoperative pRBC transfusions. This tool could be used to provide decision support for preoperative type and screen and blood orders.

The model demonstrated a comparable predictive accuracy relative to 3 machine learning–derived instruments that we created based on the same dataset. Successful implementation of machine learning to support clinical workflows requires collaboration between computer scientists and clinicians,^10^ which limits implementation. By contrast, our model can be calculated everywhere manually, which adds value to clinical medicine.

The instrument adds practical value to the care of patients undergoing operations that carry a high risk for pRBC transfusion.^18^ At our institution, we have already implemented the score for liver transplant operations where the surgical team, based on the score value, determines the number of pRBC transfusions that need to be crossmatched preoperatively. We are currently in the process of implementing the score for decision support in the electronic medical record such that the surgical team will see the predicted pRBC transfusion risk by the time they prepare their preoperative surgical orders, similar to previous quality improvement projects.^21,22^

Expectedly, preoperative anemia was the strongest predictor of intraoperative pRBC transfusion. In our population, patients with a preoperative hemoglobin less than or equal to 7.5 g/dL had the highest prediction score for an intraoperative pRBC transfusion. Even mild preoperative anemia (≤12 g/dL in female patients; ≤13 g/dL in male patients) predicted intraoperative pRBC transfusion in our study cohort, which has been reported previously.^23,24^

In a 2023 observational study in patients undergoing cardiac surgery, the authors reported a median preoperative Hb level of 12.2 g/dL in the transfused group,^25^ indicating that documented anemia is not always present by the time of pRBC transfusion. During a massive intraoperative hemorrhage, clinicians administer fluids, vasopressors, and pRBC transfusions in parallel without previous documentation of anemia. Our data demonstrate that the vast majority of patients who received pRBC without substantial anemia were hemodynamically unstable.^26,27^

We found that nonambulatory surgery (inpatients and those admitted on the day of surgery) was a strong predictor of intraoperative pRBC transfusion. This is intuitive, because pRBC transfusions are regularly used in patients undergoing more complex procedures,^18^ which should not regularly be planned in an outpatient setting.^28^

Surgical complexity defined by a high count of work relative value units and long duration of surgery were risk factors for pRBC transfusion, which has been reported previously.^29^ We observed that cardiopulmonary bypass operations were the surgery type with the highest score value in our model. In the TRICS III (Transfusion Requirements in Cardiac Surgery) trial, 52% to 72% of patients were transfused with at least 1 unit of pRBC regardless of the transfusion strategy.^30^ We also identified off-pump surgical procedures as a risk factor of pRBC transfusion. In accordance, 50.7% of the off-pump cases received at least 1 unit of pRBC vs 63.3% of the on-pump cases.^31^

Visceral transplant operations, encompassing liver, kidney, pancreas, and multivisceral transplantations, were also an independent predictor of intraoperative pRBC transfusion.^32^ Our instrument provided specific PPV and NPV for abdominal surgical procedures with high bleeding risk, such as splenectomy, pancreatectomy, retroperitoneal tumor resection, and nephrectomy.^18,33,34^ We observed that obstetric or gynecological surgery was an independent predictor of pRBC transfusion, which is in accordance with recent data.^34^

We found redo surgery to be associated with an increased risk of requiring a pRBC transfusion during surgery. Alghamdi et al^9^ included previous cardiac surgery in their TRUST score for intraoperative pRBC transfusion for patients undergoing cardiac surgery. Our score extends this applicability for a wide range of cardiac and noncardiac surgical procedures. Furthermore, emergency cases or those that started outside of regular working hours were predictive for pRBC transfusion in our score. Similar findings were demonstrated in a retrospective review of American College of Surgeons' National Surgical Quality Improvement Program data from 3 academic centers by Medvecz et al^35^; that study found that pRBC transfusions occurred in more than 11.5% of emergent operations vs 3.7% in nonemergent operations.^35^

We found that high ASA status grade along with other comorbidities in our study cohorts were predictors of intraoperative pRBC transfusion. Zhang et al^36^ have incorporated ASA status in a recently published prediction model of perioperative pRBC transfusion, and Hart et al^37^ have shown a high correlation between ASA status grade and perioperative pRBC transfusion risk in hip replacement surgery. Moreover, our data suggest that liver disease or bilirubin levels greater than 2 mg/dL (to convert to micromoles per liter, multiply by 17.104) were associated with increased risk of intraoperative pRBC transfusion, most likely due to coagulopathy.^38^

We observed an association of hypoalbuminemia with intraoperative pRBC transfusion, which has been described before as an independent risk factor for perioperative pRBC transfusion in bariatric and orthopedic operations as well as radical nephrectomies.^39,40,41,42^ Preoperative thrombocytopenia and an elevated INR were also predictors in our score. Other studies have shown that patients with deranged platelet counts and INR values had a higher incidence of intraoperative pRBC transfusion.^43,44^

Our instrument predicted massive pRBC transfusions, defined as either 3 or 10 or more RBC units. This information can be taken into account by clinicians as they make decisions on how much blood should be ordered to the operating room.

Intraoperative pRBC transfusion prediction models have focused on single surgery types.^9,45^ Our goal was to create a prediction score that represents the full surgical bandwidth of an academic health care network including cardiac and transplant surgical procedures to widen applicability while preserving precision and accuracy. The prediction score can easily be calculated by simple addition. In cases with a very low predicted risk of intraoperative pRBC requirement, the amount of a priori issued blood components could be safely reduced.

### Limitations

This study has several limitations. We did not include intraoperative measures, such as massive blood loss, in the model to enable clinicians to calculate the score prior to surgery. The instrument was developed and validated based on the data from 2 quaternary medical centers, 2 ambulatory centers, and 3 community hospitals that are affiliated with academic centers. While the demographic characteristics of patients included in our study reflect the general US population, blood use patterns may differ in rural community hospitals. We used both internal and external validation to assess the robustness and generalizability of our prediction model. However, recurrent local validation was not conducted. Recurrent local validation,^46^ which involves periodically reassessing the model’s performance within the same institution or region over time, may improve the accuracy as patient populations and clinical practices evolve. pRBC transfusions are rare events. The clinical value of this model is magnified for surgical procedures that carry a significant bleeding risk. For those procedures,^18^ the PPV of the instrument was high, and clinicians may use the information for decision support.

## Conclusions

In summary, we developed and validated a score predicting intraoperative pRBC transfusions that can be implemented into clinical practice even in the absence of advanced IT or machine learning infrastructure. The model performed better than already established models and machine learning–derived scores.

## Notes

Supplement 1. **eMethods 1.** Data Sources **eMethods 2.** Handling Missing Data: Missing-Indicator Approach **eMethods 3.** Comparing the Dose of Vasopressors Between Patients With Different Severities of Anemia **eMethods 4.** Prediction Model Creation **eMethods 5.** Model Evaluation **eMethods 6.** Development of Machine Learning Models **eTable 1.** Description and Categorization of Outcome and Candidate Predictors **eTable 2.** Description of Comorbidities Included as Candidate Predictors **eTable 3.** Sensitivity Analysis Across Various Surgical Specialties in the Development Cohort **eTable 4.** Subgroup Definitions **eTable 5.** Baseline Characteristics of 2621 Adult Patients Undergoing Surgery by the Transplant Surgery Service From 2016 to 2021, Grouped by the Administration of Blood Products **eTable 6.** Baseline Characteristics of 7220 Adult Patients Undergoing Cardiovascular or Cardiothoracic Surgery From 2016 to 2021, Grouped by the Administration of Blood Products **eTable 7.** Baseline Characteristics of 61 499 Adult Patients Undergoing Cancer Surgery From 2016 to 2021, Grouped by the Administration of Blood Products **eTable 8.** Baseline Characteristics of 80 407 Adult Patients Undergoing Gastrointestinal Surgery From 2016 to 2021, Grouped by the Administration of Blood Products **eTable 9.** Baseline Characteristics of 26 102 Adult Patients Undergoing Surgery on Their Musculoskeletal System From 2016 to 2021, Grouped by the Administration of Blood Products **eTable 10.** Comparison of Socioeconomic Measures Across Literature, Development and External Validation Cohorts **eTable 11.** Predicted Risks of Intraoperative pRBC Transfusion for Increasing Score Values **eTable 12.** Comparison of Predictive Performance of Transfuse, Different ML Models, and TRUST Score **eFigure 1.** Predictors and Their Score Values of the TRANSFUSE Model With Missing Indicator **eFigure 2.** Performance of TRANSFUSE Score in the Model With Missing Indicator **eFigure 3.** Calibration Plot For TRANSFUSE **eFigure 4.** Vasopressor Requirements Across Different Severities of Anemia **eFigure 5.** Comparing the Predictive Ability of TRANSFUSE and TRUST **eFigure 6.** Performance of TRANSFUSE Score With Additional Predictor of Perioperative Administration of Tranexamic Acid **eFigure 7.** Predictors and Their Score Values of the TRANSFUSE Model With Perioperative Tranexamic Acid Administration
eReferences.

Supplement 2.
Data Sharing Statement
