# Supplementary material for: Development and Validation of a Risk Model to Predict Intraoperative Blood Transfusion
Source: JAMA Netw Open. 2025 Apr 17;8(4):e255522. doi: 10.1001/jamanetworkopen.2025.5522 (PMC12006869; doi:10.1001/jamanetworkopen.2025.5522)
Supplement: Supplement 1. — eMethods 1. Data Sources eMethods 2. Handling Missing Data: Missing-Indicator Approach eMethods 3. Comparing the Dose of Vasopressors Between Patients With Different Severities of Anemia eMethods 4. Prediction Model Creation eMethods 5. Model Evaluation eMethods 6. Development of Machine Learning Models eTable 1. Description and Categorization of Outcome and Candidate Predictors eTable 2. Description of Comorbidities Included as Candidate Predictors eTable 3. Sensitivity Analysis Across Various Surgical Specialties in the Development Cohort eTable 4. Subgroup Definitions eTable 5. Baseline Characteristics of 2621 Adult Patients Undergoing Surgery by the Transplant Surgery Service From 2016 to 2021, Grouped by the Administration of Blood Products eTable 6. Baseline Characteristics of 7220 Adult Patients Undergoing Cardiovascular or Cardiothoracic Surgery From 2016 to 2021, Grouped by the Administration of Blood Products eTable 7. Baseline Characteristics of 61 499 Adult Patients Undergoing Cancer Surgery From 2016 to 2021, Grouped by the Administration of Blood Products eTable 8. Baseline Characteristics of 80 407 Adult Patients Undergoing Gastrointestinal Surgery From 2016 to 2021, Grouped by the Administration of Blood Products eTable 9. Baseline Characteristics of 26 102 Adult Patients Undergoing Surgery on Their Musculoskeletal System From 2016 to 2021, Grouped by the Administration of Blood Products eTable 10. Comparison of Socioeconomic Measures Across Literature, Development and External Validation Cohorts eTable 11. Predicted Risks of Intraoperative pRBC Transfusion for Increasing Score Values eTable 12. Comparison of Predictive Performance of Transfuse, Different ML Models, and TRUST Score eFigure 1. Predictors and Their Score Values of the TRANSFUSE Model With Missing Indicator eFigure 2. Performance of TRANSFUSE Score in the Model With Missing Indicator eFigure 3. Calibration Plot For TRANSFUSE eFigure 4. Vasopressor Requirements Across Different Severitie [file jamanetwopen-e255522-s001.pdf]

Supplemental Online Content

Eyth A, Borngaesser F, Rudolph MI, et al. Development and validation of a risk model to predict intraoperative blood transfusion. *JAMA Netw Open.* 2025;8(4):e255522. doi:10.1001/jamanetworkopen.2025.5522

eMethods 1. Data Sources

eMethods 2. Handling Missing Data: Missing-Indicator Approach

eMethods 3. Comparing the Dose of Vasopressors Between Patients With Different Severities of Anemia

eMethods 4. Prediction Model Creation

eMethods 5. Model Evaluation

eMethods 6. Development of Machine Learning Models

eTable 1. Description and Categorization of Outcome and Candidate Predictors

eTable 2. Description of Comorbidities Included as Candidate Predictors

eTable 3. Sensitivity Analysis Across Various Surgical Specialties in the Development Cohort

eTable 4. Subgroup Definitions

eTable 5. Baseline Characteristics of 2621 Adult Patients Undergoing Surgery by the Transplant Surgery Service From 2016 to 2021, Grouped by the Administration of Blood Products

eTable 6. Baseline Characteristics of 7220 Adult Patients Undergoing Cardiovascular or Cardiothoracic Surgery From 2016 to 2021, Grouped by the Administration of Blood Products

eTable 7. Baseline Characteristics of 61 499 Adult Patients Undergoing Cancer Surgery From 2016 to 2021, Grouped by the Administration of Blood Products

eTable 8. Baseline Characteristics of 80 407 Adult Patients Undergoing Gastrointestinal Surgery From 2016 to 2021, Grouped by the Administration of Blood Products

eTable 9. Baseline Characteristics of 26 102 Adult Patients Undergoing Surgery on Their Musculoskeletal System From 2016 to 2021, Grouped by the Administration of Blood Products

eTable 10. Comparison of Socioeconomic Measures Across Literature, Development and External Validation Cohorts

eTable 11. Predicted Risks of Intraoperative pRBC Transfusion for Increasing Score Values

© 2025 Eyth A et al. *JAMA Network Open*.

eTable 12. Comparison of Predictive Performance of Transfuse, Different ML Models, and TRUST Score

eFigure 1. Predictors and Their Score Values of the TRANSFUSE Model With Missing Indicator

eFigure 2. Performance of TRANSFUSE Score in the Model With Missing Indicator

eFigure 3. Calibration Plot For TRANSFUSE

eFigure 4. Vasopressor Requirements Across Different Severities of Anemia

eFigure 5. Comparing the Predictive Ability of TRANSFUSE and TRUST

eFigure 6. Performance of TRANSFUSE Score With Additional Predictor of Perioperative Administration of Tranexamic Acid

eFigure 7. Predictors and Their Score Values of the TRANSFUSE Model With Perioperative Tranexamic Acid Administration

eReferences.

This supplemental material has been provided by the authors to give readers additional information about their work.

© 2025 Eyth A et al. *JAMA Network Open*.

eMethods

eMethods 1. Data Sources

Development and internal temporal validation cohort

Data ranging from January 2016 to June 2021 (development) and June 2021 to February 2023 (internal temporal validation) were extracted from the electronic data management systems at Montefiore Health System (Montefiore Medical Center and its affiliated community hospitals), a major academic quaternary healthcare network in the Bronx, New York, USA.

Data were strictly de-identified per the security level of the Information Security data management standards at Montefiore and Albert Einstein College of Medicine. Sociodemographic information, including age, sex, and body mass index, was sourced from the EPIC patient information system (2024 Epic Systems Corporation, 1979 Milky Way, Verona, Wisconsin 53593, United States of America). Additionally, data on admission type, surgical procedure, duration of surgery, hospitalization, and International Classification of Diseases (9th/10th Revision, Clinical Modification [ICD-9/10-CM]) diagnostic codes from Clarity Informatics were integrated with patient-related data. Procedure-related information, including Current Procedural Terminology (CPT) codes, was obtained from the Montefiore Health System's billing database. All analyses were conducted solely using de-identified data, securely stored in a separately locked Information Technology laboratory

External validation cohort

The validation team at Beth Israel Deaconess Medical Center (BIDMC), Boston, Massachusetts, USA included data ranging from 2008 to 2022 for external validation of the score. Deidentified data were obtained from the Anesthesia Information Management system, the Perioperative Information Management System, the case mix hospital billing database (ICD-9/10 codes); and the billing database (CPT codes).

eMethods 2. Handling Missing Data: Missing-Indicator Approach

For variables with missing data, we analyzed the pattern of missingness. We found ASA score values, underweight, workRVU, emergency status and surgery outside regular working hours, as well as estimated duration of surgery greater than 120min and admission status to be missing at random.
For the primary analysis, missing data were addressed using multiple imputation.[1] As a sensitivity analysis, we applied the missing-indicator method.[2] For continuous variables, a binary indicator (1 = missing, 0 = not missing) was created, and missing values were imputed using the cohort mean or median.[2] For categorical/binary variables, a separate "missing" category was introduced.[2] Both the imputed values and missing indicators were included in the model. The prediction tool was then developed using the complete dataset, with predictors identified as shown in eTable S3. The predictors identified using the missing-indicator method were similar to those in the original model; however, the accuracy of the model derived from the missing-indicator method was lower compared to the original model derived from multiple imputation(Area under the receiver operator curve [95% confidence interval]: 0.91[0.90 to 0.91] , positive predictive value [95% confidence interval]: 8.1 (7.9 to 8.4), NPV: 99.7 [99.6 to 99.7]; eFigure 2).

© 2025 Eyth A et al. *JAMA Network Open*.

eMethods 3. Comparing the Dose of Vasopressors Between Patients With Different Severities of Anemia

We performed a two-sample t-test to compare the dose of norepinephrine equivalent between patients with different severities of anemia. Anemia was defined based on laboratory results of hemoglobin (Hb) and sex: 1) no anemia (Hb ≥12 g/dl for women and Hb ≥13g/dl for men; 2) mild preoperative anemia (Hb < 12g/dl for women and <13g/dl for men); 3) moderate anemia (Hb 7.5g/dl to 10g/dl); 4) severe anemia (Hb <7.5g/dl).

For this analysis we categorized anemia in two groups: the first group constituted patients with no or mild anemia (n=1,592) and the second group included patients with moderate or severe anemia (n=2,439). Patients with no/mild anemia received a significantly higher cumulative dose (mean ± standard deviation: 1.04±0.17 mg) of norepinephrine equivalent than the group with moderate/severe anemia (0.66±0.06 mg, *P*=.014, respectively. Among patients reveiving pRBC transfusions, we found that significantly higher doses of vasopressors compared to those that did not receive pRBC (0.80±0.08 mg versus 0.10±0.01 mg *P*<.001)

Various vasopressors are used intraoperatively at our institution. To address this, norepinephrine equivalent was calculated using the following formula: Intraoperative Norepinephrine equivalents = Intraoperative Epinephrine dose in mg + Intraoperative Norepinephrine dose in mg + (Intraoperative Phenylephrine dose in mg/10) + (Intraoperative Dopamine dose in ($mg*kg^{-1}$)/2).[3]

© 2025 Eyth A et al. *JAMA Network Open*.

eMethods 4. Prediction Model Creation

For model creation, we used stepwise backward regression to identify outcome predictors with a threshold p-value of <0.01 to retain predictors with significant associations with the outcome. Bootstrapping with 1000 samples was conducted to confirm the robustness of predictors, and penalized maximum likelihood estimation was applied to address potential overfitting of the model.[4–6] All predictors in the final model were assigned weighting values by dividing each predictor's respective beta coefficient by the smallest beta coefficient among all final predictors. These values were then rounded to the nearest integer and multiplied by the corresponding predictor's value to estimate the final prediction score (Fig 2 in main manuscript, eTable 13 in supplemental material).

eMethods 5. Model Evaluation

C-Statistic was used to assess model discrimination, while the Brier score was calculated to evaluate the model's accuracy. Further, a calibration plot was generated to assess model calibration. The TRANSFUSE (TRANSfusion Forecast Utility for Surgical Events) score was calculated by adding together all the final predictors, with each predictor multiplied by its assigned weight. In the regression model, the score was used as the only independent variable, while intraoperative pRBC transfusion was the dependent variable.
The Youden index was used to determine the optimal cut-off for dichotomizing the score between low-risk and high-risk patients. Sensitivity, specificity, and positive and negative predictive values were then assessed at this cut point.

eMethods 6. Development of Machine Learning Models

We developed three machine learning models for the development cohort to predict intraoperative transfusion of pRBC: 1) logistic regression 2) decision tree and 3) XGBoost classification model. We accounted for imbalance in data using balanced class weight to enhance model creation.

We further used Grid Search for hyperparameter tuning, and optimized models based on the F1 score:

1. Logistic regression was tuned by varying the inverse regularization parameter C across 0.01, 0.1, 1, 10, 100.
2. Decision tree was optimized for maximum depth (10, 12, 15, 20) and minimum samples split (2, 5, 10).
3. XGBoost tuning included learning rate (0.01, 0.1, 0.2), number of estimators (100, 200, 300), and maximum depth (3, 6, 9).

To quantify model uncertainty, we estimated confidence intervals for performance metrics using bootstrapping. A 95% confidence interval for accuracy was obtained by resampling test predictions multiple times and computing percentile ranges.

Our final model parameters after grid search and model performance are displayed in eTable S12.

© 2025 Eyth A et al. *JAMA Network Open*.

eTable 1. Description and Categorization of Outcome and Candidate Predictors

	**Definition**	**Categorization**
**Outcome**		
Intraoperative pRBC transfusion	Transfusion of at least one pRBC within surgery start and surgery end time	Y/N
**Candidate predictors**		
Demographics		
Age over 75 years	At admission	Y/N
Female sex	Sex assigned at birth	Y/N
Underweight	Body mass index (BMI) below 18.5	Y/N
High ASA Status	ASA >2; as recorded in the electronic health record	Y/N
Comorbidities/Medication		
Anticoagulation	Prescription of anticoagulation within 2 years before surgery consisting of direct Factor Xa inhibitors (apixaban, eliquis, rivaroxaban, xarelto, edoxaban, savaysa, betrixaban, bevyxxa), direct thrombin inhibitors (argatroban, acova, dabigatran, pradaxa), and vitamin K-dependent antagonists (coumadin, warfarin)[9]	Y/N
Antiplatelets	Prescription of antiplatelets within 2 years before surgery (acetylsalicylic acid, clopidogrel, prasugrel, ticagrelor, ticlopidine, cangrelor, cilostazol, abciximab, eptifibatide, dipyridamole)[9]	Y/N
Renal failure	History of comorbidity within one year prior to surgery, defined as detailed in eTable S2<br>OR the most recent GFR within 1 year before surgery <15ml/min	Y/N
Liver disease	History of comorbidity within one year prior to surgery, defined as detailed in eTable S2<br>OR preoperative bilirubin ³2.0mg/dl	Y/N
Mild Anemia	History of comorbidity within one year prior to surgery, defined as detailed in eTable S2, if no preoperative hemoglobin was assessed<br>OR preoperative hemoglobin <12g/dl and ³10g/dl in females<br>OR preoperative hemoglobin <13g/dl and ³10g/dl in males	Y/N
Moderate Anemia	Preoperative hemoglobin of <10g/dl and >7.5g/dl	Y/N
Severe Anemia	Preoperative hemoglobin of £7.5g/dl	Y/N
Hypoalbuminemia	Preoperative albumin of <3.5g/dl	Y/N
Thrombocytopenia	Preoperative platelet count of <150 000/ml	Y/N
Abnormal INR	Preoperative INR of >1.2 and £2.0	Y/N
Surgery type		
Cardiopulmonary bypass	Procedure performed on cardiopulmonary bypass, as noted in the electronic health record, more detailed information in eTable S3	Y/N

© 2025 Eyth A et al. *JAMA Network Open*.

Cardiac off-pump surgery	Procedure performed on the respective organs, excluding procedures done on cardiopulmonary bypass, more detailed information in eTable S3	Y/N
Thoracic off-pump surgery	Procedure performed on the respective organs, excluding procedures done on cardiopulmonary bypass, more detailed information in eTable S3	Y/N
Vascular surgery	Procedure performed on the respective organs, more detailed information in eTable S3	Y/N
Orthopedic surgery	Procedure performed on the respective organs, more detailed information in eTable S3	Y/N
Neurosurgery	Procedure performed on the respective organs, more detailed information in eTable S3	Y/N
Abdominal surgery	Procedure performed on the respective organs, more detailed information in eTable S3	Y/N
Visceral transplant	Procedure performed on the respective organs, more detailed information in eTable S3	Y/N
Plastic surgery	Procedure performed on the respective organs, more detailed information in eTable S3	Y/N
Urological surgery	Procedure performed on the respective organs, more detailed information in eTable S3	Y/N
Obstetric/Gynecological surgery	Procedure performed on the respective organs, more detailed information in eTable S3	Y/N
Redo-surgery	Revision or redo of a procedure that was performed in the same patient at the same organ any time before, more detailed information in eTable S3	Y/N
Other preoperative information		
Emergency status	Emergency status as recorded in electronic health record or procedure started outside of regular working hours	Y/N
High procedural complexity	Defined as higher than median work relative value units based on CPT^{Ò}	Y/N
Long estimated surgical duration	Estimated surgical duration of ^{3}120min as recorded in electronic health record	Y/N
Non-Ambulatory Surgery	Defined as patients admitted on the day of their procedure OR at least one day before the surgery	Y/N

Abbreviations: pRBC – packed red blood cells, BMI – Body mass index, ASA – American Society of Anesthesiologists, GFR - glomerular filtration rate, INR - International normalized ratio, CPT^{Ò} – Current Procedural Terminology

© 2025 Eyth A et al. *JAMA Network Open*.

eTable 2. Description of Comorbidities Included as Candidate Predictors

Variable	ICD-9	ICD-10
Anemia	[280.X-285.X]	[D50.X-D64.X]
Liver disease	[456.0X-456.2X], [572.2-572.8] 070.22, 070.23, 070.32, 070.33, 070.44, 070.54, 070.6, 070.9, 573.3, 573.4, 573.8, 573.9, [570.X-572]	I85.0, I85.9, I86.4, I98.2, K70.4, K71.1, K72.1, K72.9, [K76.5X-K76.7X], I85.0, I85.9, I86.4, I98.2, K70.4, K71.1, K72.1, K72.9, [K76.5X-K76.7X] V42.7, B18, [K70.0 – K70.3], K70.9, [K71.3 - K71.5], K71.7, K73, K74, K76.0, K76, K76.3, K76.4, K76.8, K76.9, Z94.4
Renal Failure	403.01, 403.11, 403.91, 404.02, 404.03, 404.12, 404.13, 404.92, 404.93, 582.X, 583.X, 585.X, 586.X, 588.0, V42.0, V45.1, V56.X	I12.0, I13.1, [N03.2X-N03.7X], [N05.2X-N05.7X], N18.X, N19.X, N25.0, [Z49.0X-Z49.2X], Z94.0, Z99.2

Abbreviations: ICD- International classification of diseases

© 2025 Eyth A et al. *JAMA Network Open*.

eTable 3. Sensitivity Analysis Across Various Surgical Specialties in the Development Cohort

Subgroup	N	Incidence of intraoperative pRBC transfusion N (%)	ROC AUC (95% CI)
**Cancer Surgery**	61,499	2,100 (3.41%)	0.87 (0.87-0.88)
**Cardiac Surgery**	7,220	935 (12.95%)	0.83 (0.81-0.84)
**Gastrointestinal Surgery**	80,407	802 (1.00%)	0.96 (0.96-0.97)
**Musculoskeletal Surgery**	26,102	763 (2.92%)	0.87 (0.85-0.88)
**Transplant Surgery**	2,621	300 (11.45%)	0.86 (0.84-0.88)

Abbreviations: pRBC-packed red blood cells, ROC AUC– Receiver operator curve area under the curve,

© 2025 Eyth A et al. *JAMA Network Open*.

eTable 4. Subgroup Definitions

Subgroup	Definition
Transplant surgery	Surgical service as noted in the electronic health record.
Cardiac surgery	Surgical service as noted in the electronic health record.
Cancer surgery	Patients with a solid cancer diagnosis, identified using International Classification of Disease, 9th (ICD-9) and 10th (ICD-10) revision diagnostic codes, undergoing a cancer resection procedure, identified through ICD-9 and ICD-10 procedure and CPTÒ codes for the surgery specific to the corresponding cancer entity, as identified through ICD-9 and ICD-10 diagnostic codes.[10]
Gastrointestinal surgery	Patients undergoing a surgery with the CPTÒ 40490–49999
Surgery on the musculoskeletal system	Patients undergoing a surgery with the CPTÒ 20000–29999

Abbreviations: ICD- International classification of diseases, CPT- Current Procedural Terminology

eTable 5. Baseline Characteristics of 2621 Adult Patients Undergoing Surgery by the Transplant Surgery Service From 2016 to 2021, Grouped by the Administration of Blood Products

	**No intraoperative Transfusion N=2,321**	**Intraoperative Transfusion N=300**
Age	54.0 ± 14.9	54.3 ± 12.9
Female sex	967 (41.7%)	125 (41.7%)
Underweight, BMI<18.5	43 (1.9%)	10 (3.3%)
High ASA Status, >2	1,925 (82.9%)	298 (99.3%)
Emergency surgery or surgery started outside of regular working hours	609 (26.2%)	182 (60.7%)
High surgical complexity	1,842 (79.4%)	292 (97.3%)
Estimated surgical duration >120min	1,906 (82.1%)	292 (97.3%)
Mild anemia	966 (41.6%)	81 (27.0%)
Moderate anemia	550 (23.7%)	139 (46.3%)
Severe anemia	105 (4.5%)	59 (19.7%)
Liver disease, Bilirubin ≥2mg/dl	761 (32.8%)	228 (76.0%)
Renal failure, GFR <15	1,421 (61.2%)	161 (53.7%)
Low preoperative Albumin, <3.5g/dl	150 (6.5%)	80 (26.7%)
Low preoperative Platelet count, <150,000/ml	499 (21.5%)	173 (57.7%)
High preoperative INR ratio, >1.2 and <2.0	208 (9.0%)	104 (34.7%)
Redo Surgery	57 (2.5%)	10 (3.3%)
Surgery on Cardiopulmonary Bypass	0 (0.0%)	0 (0.0%)
Obstetric/Gynecological Surgery	0 (0.0%)	0 (0.0%)
Urological Surgery	190 (8.2%)	4 (1.3%)
Cardiac Surgery off pump	0 (0.0%)	0 (0.0%)
Thoracic Surgery off pump	1 (0.0%)	0 (0.0%)
Vascular Surgery	124 (5.3%)	0 (0.0%)
Orthopedic Surgery	1 (0.0%)	0 (0.0%)
Neurosurgery	0 (0.0%)	0 (0.0%)
Major Abdominal Surgery	1,077 (46.4%)	95 (31.7%)
Visceral Transplant Surgery	808 (34.8%)	193 (64.3%)
Plastic Surgery	2 (0.1%)	0 (0.0%)
Non-Ambulatory Surgery	1,945 (83.8%)	300 (100.0%)

Data are presented as mean ± SD for continuous measures, and n (%) for categorical measures.
Abbreviations: BMI – Body mass index, CPT© – Current procedural terminology, GFR - glomerular filtration rate, INR - International normalized ratio

© 2025 Eyth A et al. *JAMA Network Open*.

eTable 6. Baseline Characteristics of 7220 Adult Patients Undergoing Cardiovascular or Cardiothoracic Surgery From 2016 to 2021, Grouped by the Administration of Blood Products

	**No intraoperative Transfusion N=6,285**	**Intraoperative Transfusion N=935**
Age	62.8 ± 15.0	61.6 ± 13.7
Female sex	2,429 (38.6%)	437 (46.7%)
Underweight, BMI<18.5	104 (1.7%)	23 (2.5%)
High ASA Status, >2	5,921 (94.2%)	926 (99.0%)
Emergency surgery or surgery started outside of regular working hours	1,572 (25.0%)	414 (44.3%)
High surgical complexity	4,929 (78.4%)	885 (94.7%)
Estimated surgical duration >120min	5,326 (84.7%)	922 (98.6%)
Mild anemia	2,520 (40.1%)	341 (36.5%)
Moderate anemia	890 (14.2%)	414 (44.3%)
Severe anemia	73 (1.2%)	79 (8.4%)
Liver disease, Bilirubin ≥2mg/dl	1,033 (16.4%)	269 (28.8%)
Renal failure, GFR <15	1,650 (26.3%)	423 (45.2%)
Hypoalbuminemia	263 (4.2%)	102 (10.9%)
Thrombocytopenia	842 (13.4%)	254 (27.2%)
Abnormal preoperative INR	723 (11.5%)	195 (20.9%)
Redo Surgery	166 (2.6%)	77 (8.2%)
Surgery on Cardiopulmonary Bypass	2,415 (38.4%)	573 (61.3%)
Obstetric/Gynecological Surgery	1 (0.0%)	0 (0.0%)
Urological Surgery	0 (0.0%)	0 (0.0%)
Cardiac Surgery off pump	1,564 (24.9%)	178 (19.0%)
Thoracic Surgery off pump	1,143 (18.2%)	117 (12.5%)
Vascular Surgery	257 (4.1%)	70 (7.5%)
Orthopedic Surgery	2 (0.0%)	0 (0.0%)
Neurosurgery	7 (0.1%)	0 (0.0%)
Major Abdominal Surgery	15 (0.2%)	3 (0.3%)
Visceral Transplant Surgery	0 (0.0%)	1 (0.1%)
Plastic Surgery	42 (0.7%)	7 (0.7%)
Non-Ambulatory Surgery	5,901 (93.9%)	930 (99.5%)

Data are presented as mean ± SD for continuous measures, and n (%) for categorical measures.
Abbreviations: BMI – Body mass index, CPT^Ò– Current procedural terminology, GFR - glomerular filtration rate, INR - International normalized ratio

© 2025 Eyth A et al. *JAMA Network Open*.

eTable 7. Baseline Characteristics of 61 499 Adult Patients Undergoing Cancer Surgery From 2016 to 2021, Grouped by the Administration of Blood Products

	**No intraoperative Transfusion N=59,399**	**Intraoperative Transfusion N=2,100**
Age	57.5 ± 17.0	60.9 ± 16.2
Female sex	35,214 (59.3%)	1,124 (53.5%)
Underweight, BMI<18.5	1,268 (2.1%)	67 (3.2%)
High ASA Status, >2	36,673 (61.7%)	1,772 (84.4%)
Emergency surgery or surgery started outside of regular working hours	10,072 (17.0%)	719 (34.2%)
High surgical complexity	40,869 (68.8%)	1,873 (89.2%)
Estimated surgical duration >120min	39,694 (66.8%)	1,871 (89.1%)
Mild anemia	18,968 (31.9%)	539 (25.7%)
Moderate anemia	11,360 (19.1%)	953 (45.4%)
Severe anemia	1,760 (3.0%)	374 (17.8%)
Liver disease, Bilirubin ≥2mg/dl	9,926 (16.7%)	594 (28.3%)
Renal failure, GFR <15	12,903 (21.7%)	802 (38.2%)
Hypoalbuminemia	2,911 (4.9%)	269 (12.8%)
Thrombocytopenia	4,807 (8.1%)	424 (20.2%)
Abnormal preoperative INR	4,075 (6.9%)	408 (19.4%)
Redo Surgery	1,399 (2.4%)	138 (6.6%)
Surgery on Cardiopulmonary Bypass	71 (0.1%)	18 (0.9%)
Obstetric/Gynecological Surgery	2,729 (4.6%)	182 (8.7%)
Urological Surgery	2,122 (3.6%)	87 (4.1%)
Cardiac Surgery off pump	242 (0.4%)	90 (4.3%)
Thoracic Surgery off pump	1,154 (1.9%)	81 (3.9%)
Vascular Surgery	6,260 (10.5%)	319 (15.2%)
Orthopedic Surgery	15,677 (26.4%)	565 (26.9%)
Neurosurgery	2,932 (4.9%)	130 (6.2%)
Major Abdominal Surgery	16,101 (27.1%)	533 (25.4%)
Visceral Transplant Surgery	100 (0.2%)	110 (5.2%)
Plastic Surgery	890 (1.5%)	33 (1.6%)
Non-Ambulatory Surgery	59,000 (99.3%)	2,097 (99.9%)

Data are presented as mean ± SD for continuous measures, and n (%) for categorical measures.
Abbreviations: BMI – Body mass index, CPT^{Ò}– Current procedural terminology, GFR - glomerular filtration rate, INR - International normalized ratio

© 2025 Eyth A et al. *JAMA Network Open*.

eTable 8. Baseline Characteristics of 80 407 Adult Patients Undergoing Gastrointestinal Surgery From 2016 to 2021, Grouped by the Administration of Blood Products

	**No intraoperative Transfusion N=79,605**	**Intraoperative Transfusion N=802**
Age	57.1 ± 16.5	61.5 ± 16.1
Female sex	46,007 (57.8%)	365 (45.5%)
Underweight, BMI<18.5	1,659 (2.1%)	40 (5.0%)
High ASA Status, >2	44,309 (55.7%)	741 (92.4%)
Emergency surgery or surgery started outside of regular working hours	9,452 (11.9%)	416 (51.9%)
High surgical complexity	21,535 (27.1%)	656 (81.8%)
Estimated surgical duration >120min	19,546 (24.6%)	682 (85.0%)
Mild anemia	19,923 (25.0%)	162 (20.2%)
Moderate anemia	11,988 (15.1%)	371 (46.3%)
Severe anemia	2,439 (3.1%)	214 (26.7%)
Liver disease, Bilirubin ≥2mg/dl	16,184 (20.3%)	388 (48.4%)
Renal failure, GFR <15	13,263 (16.7%)	302 (37.7%)
Hypoalbuminemia	3,519 (4.4%)	170 (21.2%)
Thrombocytopenia	7,554 (9.5%)	287 (35.8%)
Abnormal preoperative INR	5,018 (6.3%)	242 (30.2%)
Redo Surgery	326 (0.4%)	8 (1.0%)
Surgery on Cardiopulmonary Bypass	0 (0.0%)	0 (0.0%)
Obstetric/Gynecological Surgery	79 (0.1%)	7 (0.9%)
Urological Surgery	63 (0.1%)	7 (0.9%)
Cardiac Surgery off pump	3 (0.0%)	1 (0.1%)
Thoracic Surgery off pump	84 (0.1%)	0 (0.0%)
Vascular Surgery	68 (0.1%)	4 (0.5%)
Orthopedic Surgery	18 (0.0%)	0 (0.0%)
Neurosurgery	1 (0.0%)	0 (0.0%)
Major Abdominal Surgery	23,912 (30.0%)	514 (64.1%)
Visceral Transplant Surgery	90 (0.1%)	131 (16.3%)
Plastic Surgery	83 (0.1%)	6 (0.7%)
Non-Ambulatory Surgery	39,539 (49.7%)	800 (99.8%)

Data are presented as mean ± SD or median (IQR) for continuous measures, and n (%) for categorical measures.
*Abbreviations: BMI – Body mass index, CPT*Ò*– Current procedural terminology, GFR - glomerular filtration rate, INR - International normalize*

© 2025 Eyth A et al. *JAMA Network Open*.

eTable 9. Baseline Characteristics of 26 102 Adult Patients Undergoing Surgery on Their Musculoskeletal System From 2016 to 2021, Grouped by the Administration of Blood Products

	**No intraoperative Transfusion N=25,339**	**Intraoperative Transfusion N=763**
Age	58.3 ± 16.1	63.7 ± 16.8
Female sex	14,987 (59.1%)	405 (53.1%)
Underweight, BMI<18.5	392 (1.5%)	32 (4.2%)
High ASA Status, >2	13,662 (53.9%)	639 (83.7%)
Emergency surgery or surgery started outside of regular working hours	2,276 (9.0%)	142 (18.6%)
High surgical complexity	16,621 (65.6%)	700 (91.7%)
Estimated surgical duration >120min	18,202 (71.8%)	657 (86.1%)
Mild anemia	7,158 (28.2%)	190 (24.9%)
Moderate anemia	3,236 (12.8%)	387 (50.7%)
Severe anemia	291 (1.1%)	100 (13.1%)
Liver disease, Bilirubin ≥2mg/dl	2,306 (9.1%)	149 (19.5%)
Renal failure, GFR <15	5,522 (21.8%)	321 (42.1%)
Hypoalbuminemia	723 (2.9%)	92 (12.1%)
Thrombocytopenia	1,305 (5.2%)	114 (14.9%)
Abnormal preoperative INR	1,031 (4.1%)	116 (15.2%)
Redo Surgery	1,054 (4.2%)	104 (13.6%)
Surgery on Cardiopulmonary Bypass	0 (0.0%)	0 (0.0%)
Obstetric/Gynecological Surgery	5 (0.0%)	2 (0.3%)
Urological Surgery	4 (0.0%)	0 (0.0%)
Cardiac Surgery off pump	19 (0.1%)	1 (0.1%)
Thoracic Surgery off pump	237 (0.9%)	32 (4.2%)
Vascular Surgery	3,034 (12.0%)	179 (23.5%)
Orthopedic Surgery	21,091 (83.2%)	652 (85.5%)
Neurosurgery	1,302 (5.1%)	51 (6.7%)
Major Abdominal Surgery	29 (0.1%)	0 (0.0%)
Visceral Transplant Surgery	0 (0.0%)	0 (0.0%)
Plastic Surgery	232 (0.9%)	19 (2.5%)
Non-Ambulatory Surgery	19,138 (75.5%)	763 (100.0%)

Data are presented as mean ± SD for continuous measures, and n (%) for categorical measures.
Abbreviations: BMI – Body mass index, CPT®– Current procedural terminology, GFR - glomerular filtration rate, INR - International normalized ratio

© 2025 Eyth A et al. *JAMA Network Open.*

eTable 10. Comparison of Socioeconomic Measures Across Literature, Development and External Validation Cohorts

	Goel et al 2018 (ACS-NSQIP)[11]	Chervu et al, 2024 NIS[12]	Development cohort MMC	External validation cohort BIDMC
Median Age (years)	58	64	59	57
60 years and older	43.3%	n/a	49.1%	45.7%
Sex assigned at birth				
Female	56.8%	53.6%	60.1%	57.1%
Male	43.2%	46.4	39.9%	42.9%
Race/Ethnicity				
Non-Hispanic White	73.5%	73.2%	14.5%	68.8%
Non-Hispanic Black	10.1%	11.2%	29.4%	10.0%
Hispanic	n/a	8.3%	38.8%	5.2%
Non-Hispanic Asian	2.8%	4.9%	2.6%	4.9%
Others	1.1%		7.1%	11.1%
Unknown/ declined	12.5%	n/a	7.6%	0.0%

Race/Ethnicity were based on the patients self-identification, as given in the charts. This definition adheres to the CDC categorization.[13]
Abbreviations: n/a — not available; NSQIP — American College of Surgery National Surgical Quality Improvement Program; NIS – – National (Nationwide) Inpatient Sample; MMC — Montefiore Medical Center; BIDMC — Beth Israel Deaconess Medical Center

© 2025 Eyth A et al. *JAMA Network Open*.

eTable 11. Predicted Risks of Intraoperative pRBC Transfusion for Increasing Score Values

This table is showing the predicted risk of intraoperative pRBC transfusion for increasing score values of TRANSFUSE as estimated in the development cohort.

Score point value	Predicted risk of intraoperative pRBC transfusion in per cent (%)	95% Confidence interval (CI)
0	0.000027	0.000022–0.000031
1	0.000033	0.000027–0.000039
2	0.000041	0.000034–0.000049
3	0.000052	0.000043–0.000061
4	0.000065	0.000054–0.000076
5	0.000081	0.000068– 0.000094
6	0.000101	0.000085–0.000117
7	0.000127	0.000107–0.000146
8	0.000158	0.000135–0.000181
9	0.000198	0.000170–0.000226
10	0.000247	0.000214–0.000281
11	0.000309	0.000268–0.000350
12	0.000387	0.000338–0.000436
13	0.000483	0.000424–0.000542
14	0.000604	0.000534– 0.000675
15	0.000755	0.000671–0.000840
16	0.000944	0.000843–0.001046
17	0.001181	0.001059–0.001302
18	0.001476	0.001331–0.001620
19	0.001845	0.001673–0.002017
20	0.002306	0.002102–0.002510
21	0.002881	0.002640–0.003123
22	0.003600	0.003315–0.003885
23	0.004497	0.004162–0.004833
24	0.005617	0.005224–0.006011
25	0.007014	0.006553–0.007475
26	0.008755	0.008218–0.009292
27	0.010923	0.010300–0.011547
28	0.013621	0.012899–0.014344
29	0.016974	0.016141–0.017808
30	0.021135	0.020175–0.022095
31	0.026288	0.025183–0.027393
32	0.032656	0.031384–0.033928
33	0.040502	0.039031–0.041972
34	0.050135	0.048422–0.051848
35	0.061912	0.059895–0.063929
36	0.076233	0.073825–0.078641
37	0.093536	0.090619–0.096453
38	0.114282	0.110704–0.117859

© 2025 Eyth A et al. *JAMA Network Open*.

39	0.138923	0.134503–0.143342
40	0.167870	0.162402–0.173337
41	0.201438	0.194709–0.208167
42	0.239784	0.231593–0.247974
43	0.282844	0.273035–0.292653
44	0.330277	0.318764–0.341790
45	0.381434	0.368232–0.394636
46	0.435364	0.420608–0.450120
47	0.490868	0.474814–0.506923
48	0.546598	0.529606–0.563590
49	0.601184	0.583686–0.618683
50	0.653366	0.635820–0.670913
51	0.702104	0.684946–0.719262
52	0.746646	0.730255–0.763037
53	0.786553	0.771224–0.801882
54	0.821675	0.807608–0.835742
55	0.852105	0.839410–0.864799
56	0.878112	0.866821–0.889403
57	0.900082	0.890164–0.910000
58	0.918460	0.909841–0.927079
59	0.933707	0.926284–0.941129
60	0.946269	0.939926–0.952612
61	0.956562	0.951176–0.961948
62	0.964956	0.960408–0.969504

Abbreviations: pRBC – packed red blood cells

© 2025 Eyth A et al. *JAMA Network Open*.

eTable 12. Comparison of Predictive Performance of Transfuse, Different ML Models, and TRUST Score

	PPV	NPV	ROC AUC (95%CI)
TRANSFUSE	8.9%	99.7%	0.93 (0.92 to 0.93)
ML logistic regression	9.4%	99.7%	0.92 (0.92 to 0.93)
ML XGBoost	8.1%	99.7%	0.92 (0.91 to 0.92)
ML Random Forest	11.2%	99.4%	0.84 (0.83 to 0.85)
TRUST	2.6%	99.2%	0.64 (0.63 to 0.64)

Abbreviations: 95%CI, 95% confidence interval; ML, machine learning; NPV, negative predictive value; PPV, positive predictive value; ROC AUC, Receiver operator curve area under the curve

eFigure 1. Predictors and Their Score Values of the TRANSFUSE Model With Missing Indicator

A.

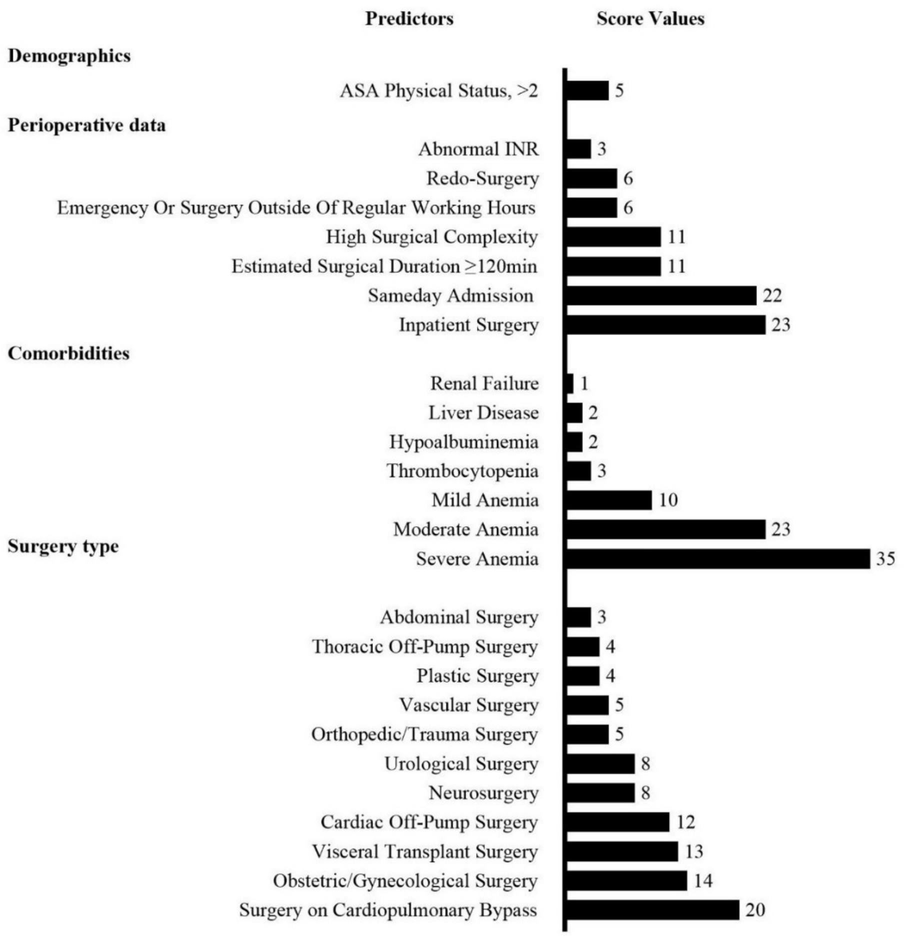

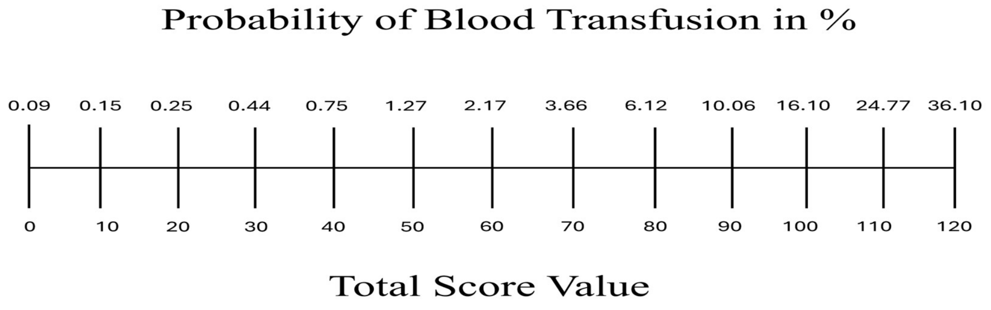

B.

A. The variables included in the TRANSFUSE model missing indicator are summarized with corresponding score point values.
B. Probability of intraoperative pRBC transfusion in percent (0.09% to 36.10%) in reference to the total score value that as patient can receive (0 to 120).

© 2025 Eyth A et al. *JAMA Network Open*.

eFigure 2. Performance of TRANSFUSE Score in the Model With Missing Indicator

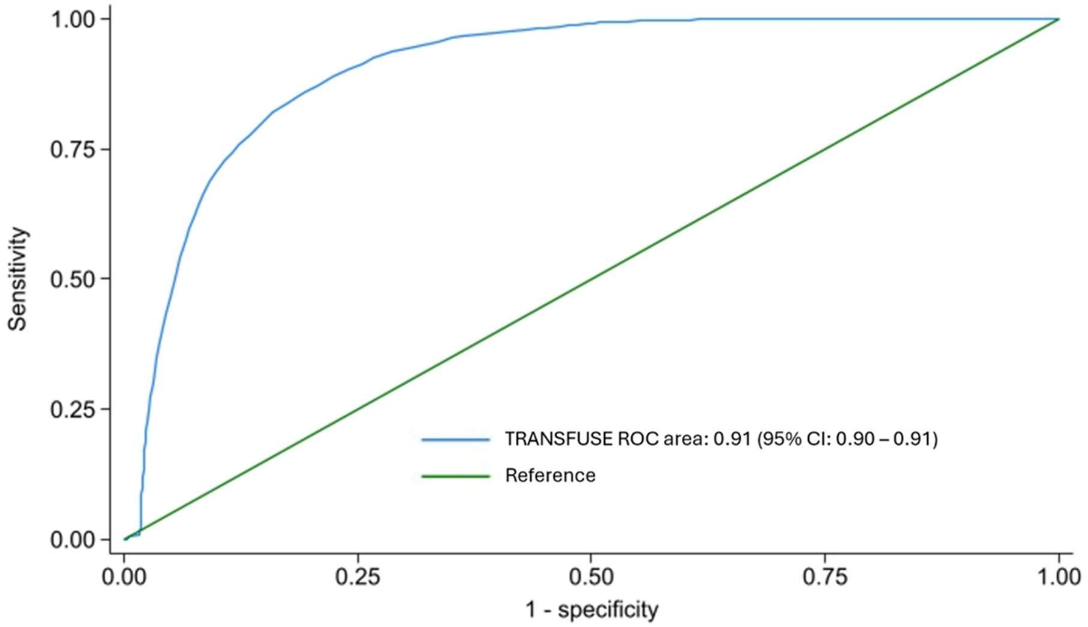

Displayed are the receiver operating curves (ROC) of the TRANSFUSE score in the model with missing indicator reflecting sensitivity versus 1-specificity. A reference line is displayed in green.

© 2025 Eyth A et al. *JAMA Network Open*.

eFigure 3. Calibration Plot For TRANSFUSE

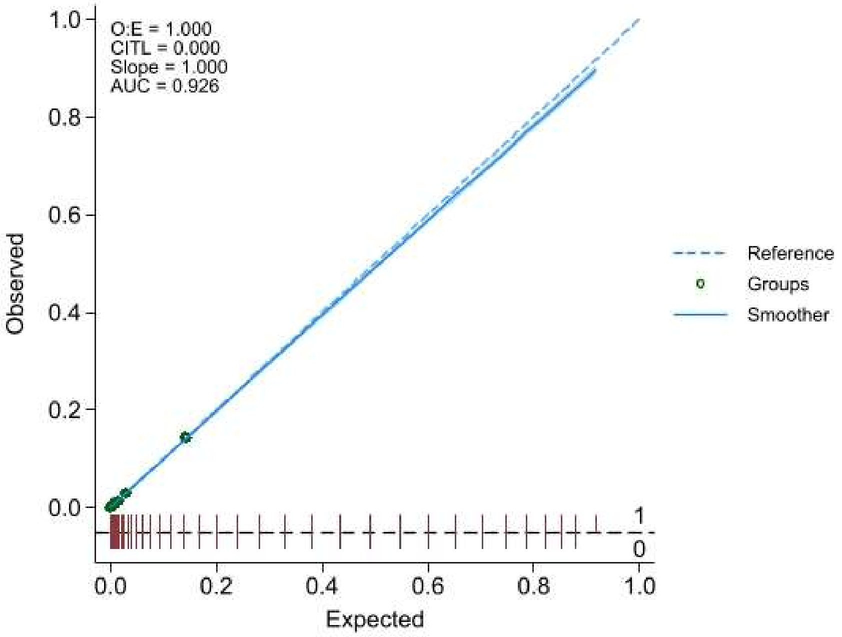

The figure demonstrates an optimal calibration

© 2025 Eyth A et al. *JAMA Network Open*.

eFigure 4. Vasopressor Requirements Across Different Severities of Anemia

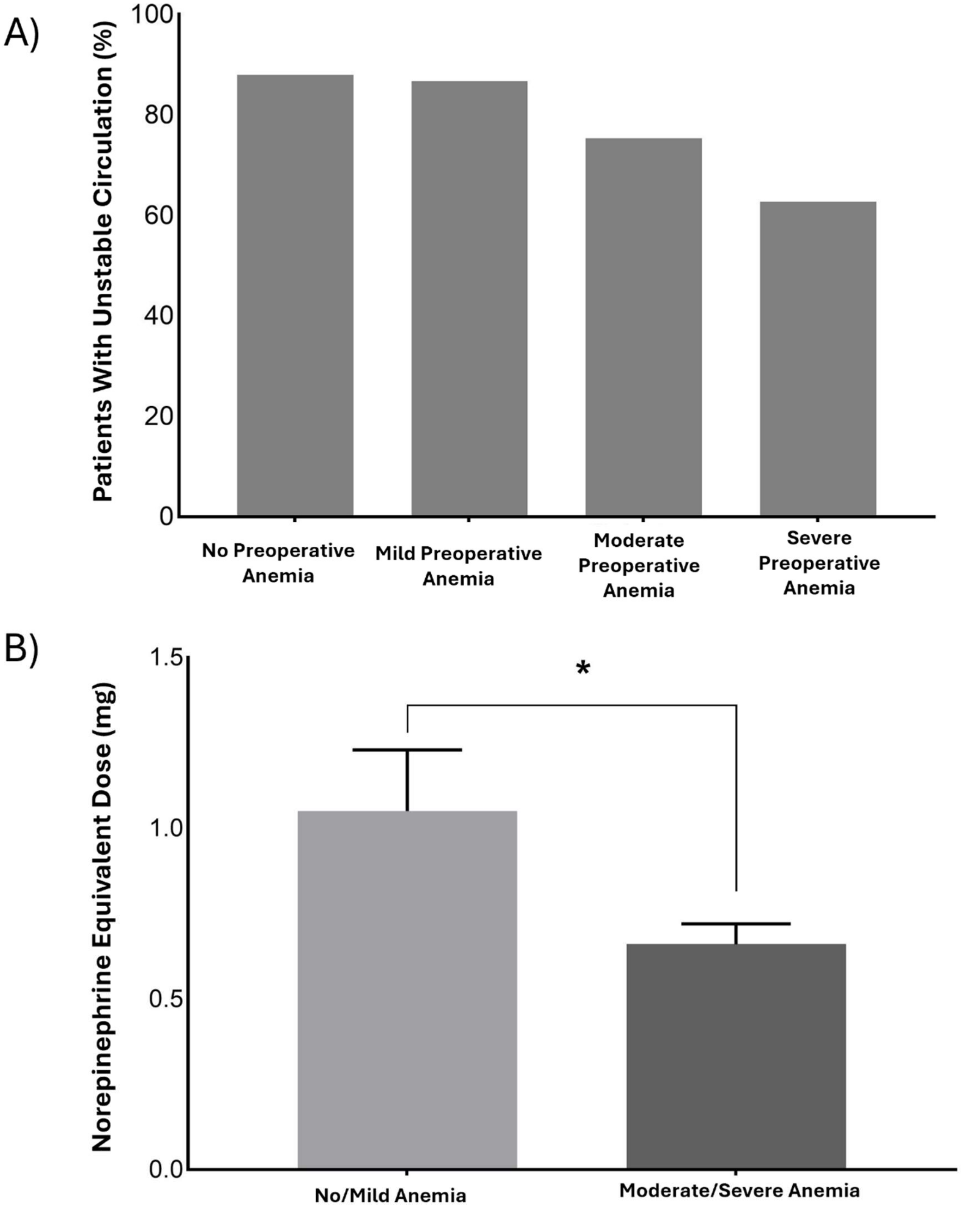

Percentage of transfused patients requiring any intraoperative vasopressors to compensate for an unstable circulation who receive pRBCs after presenting with no, mild, moderate and severe preoperative anemia. Patients who receive an intraoperative transfusion with no preoperative anemia have the highest percentage (87.9%) of intraoperative vasopressor requirements.

Cumulative intraoperative norepinephrine equivalent dose requirement in transfused patients with no/mild vs moderate/severe preoperative anemia. Mean±SEM, *=p<0.05

© 2025 Eyth A et al. *JAMA Network Open*.

eFigure 5. Comparing the Predictive Ability of TRANSFUSE and TRUST

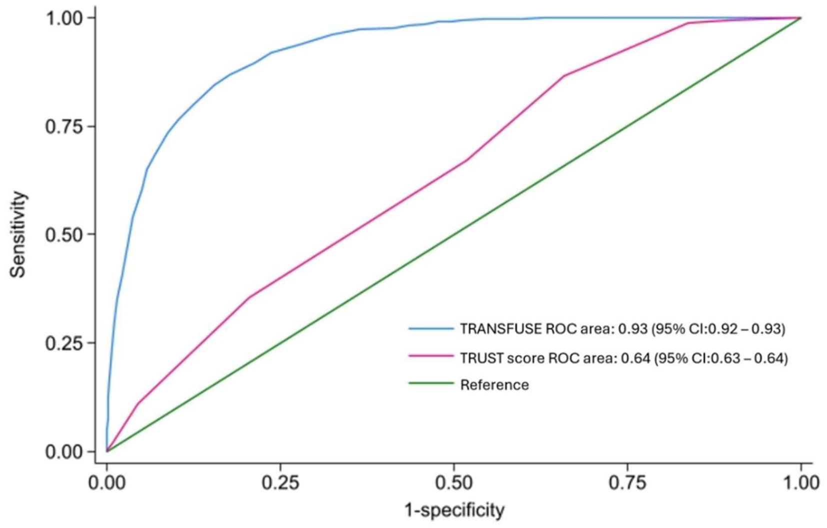

Displayed are the receiver operating curves (ROC) of the TRANSFUSE model in blue and the TRUST model in pink reflecting sensitivity versus 1-specificity. A reference line is displayed in green.
TRANSFUSE predicts intraoperative red blood cell transfusion with a higher predictive ability (area under the curve: 0.93 (95% CI 0.92 - 0.93)) compared to TRUST (area under the curve: 0.64 (95% CI:0.63 - 0.64; P<0.001)) within our study cohort.

© 2025 Eyth A et al. *JAMA Network Open*.

eFigure 6. Performance of TRANSFUSE Score With Additional Predictor of Perioperative Administration of Tranexamic Acid

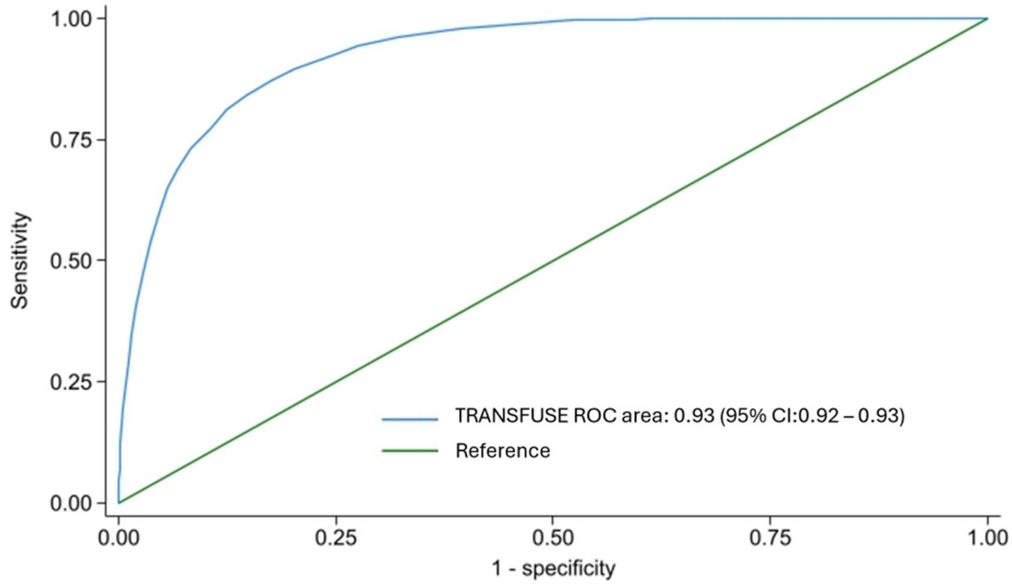

Displayed are the receiver operating curves (ROC) of the TRANSFUSE model reflecting sensitivity versus 1-specificity. A reference line is displayed in green.
TRANSFUSE predicts intraoperative red blood cell transfusion with a high predictive ability (area under the curve: 0.93 (95% CI: 0.92 – 0.93)).

© 2025 Eyth A et al. *JAMA Network Open*.

eFigure 7. Predictors and Their Score Values of the TRANSFUSE Model With Perioperative Tranexamic Acid Administration

A.

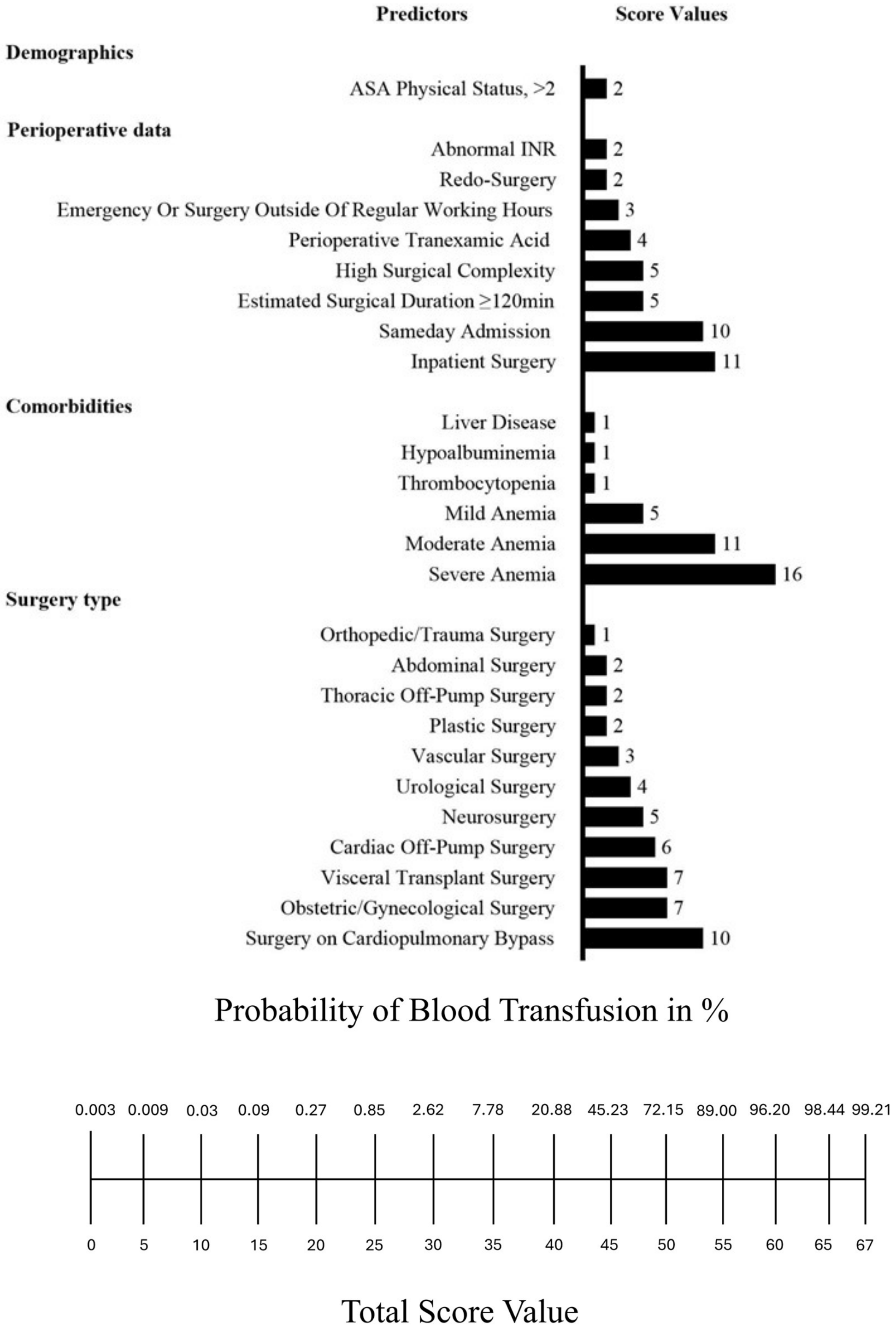

.

A. The variables included in the TRANSFUSE model missing indicator are summarized with corresponding score point values.
B. Probability of intraoperative pRBC transfusion in percent (0.003% to 99.21%) in reference to the total score value that as patient can receive (0 to 67).

© 2025 Eyth A et al. *JAMA Network Open*.

eReferences.

1. Sterne JAC, White IR, Carlin JB, et al. Multiple imputation for missing data in epidemiological and clinical research: potential and pitfalls. *BMJ*. 2009;338(jun29 1):b2393-b2393. doi:10.1136/bmj.b2393
2. Groenwold RHH, White IR, Donders ART, Carpenter JR, Altman DG, Moons KGM. Missing covariate data in clinical research: when and when not to use the missing-indicator method for analysis. *Can Med Assoc J*. 2012;184(11):1265-1269. doi:10.1503/cmaj.110977
3. Patel BM, Chittock DR, Russell JA, Walley KR. Beneficial effects of short-term vasopressin infusion during severe septic shock. *Anesthesiology*. 2002;96(3):576-582. doi:10.1097/00000542-200203000-00011
4. Platzbecker K, Grabitz SD, Raub D, et al. Development and external validation of a prognostic model for ischaemic stroke after surgery. *Br J Anaesth*. 2021;127(5):713-721. doi:10.1016/j.bja.2021.05.035
5. Lukannek C, Shaefi S, Platzbecker K, et al. The development and validation of the Score for the Prediction of Postoperative Respiratory Complications (SPORC-2) to predict the requirement for early postoperative tracheal re-intubation: a hospital registry study. *Anaesthesia*. 2019;74(9):1165-1174. doi:10.1111/anae.14742
6. Luedeke CM, Rudolph MI, Pulverenti TS, et al. Development and validation of a score for prediction of postoperative respiratory complications in infants and children (SPORC-C). *Br J Anaesth*. 2025;134(1):212-220. doi:10.1016/j.bja.2024.07.011
7. Debray TPA, Collins GS, Riley RD, et al. Transparent reporting of multivariable prediction models developed or validated using clustered data: TRIPOD-Cluster checklist. *BMJ*. Published online February 7, 2023:e071018. doi:10.1136/bmj-2022-071018
8. Husereau D, Drummond M, Augustovski F, et al. Consolidated Health Economic Evaluation Reporting Standards (CHEERS) 2022 Explanation and Elaboration: A Report of the ISPOR CHEERS II Good Practices Task Force. *Value in Health*. 2022;25(1):10-31. doi:10.1016/j.jval.2021.10.008
9. Friedrich S, Ng PY, Platzbecker K, et al. Patent foramen ovale and long-term risk of ischaemic stroke after surgery. *Eur Heart J*. 2019;40(11):914-924. doi:10.1093/eurheartj/ehy402
10. Blank M, Katsiampoura A, Wachtendorf LJ, et al. Association Between Intraoperative Dexamethasone and Postoperative Mortality in Patients Undergoing Oncologic Surgery: A Multicentric Cohort Study. *Ann Surg*. 2023;278(1):e105-e114. doi:10.1097/SLA.0000000000005526
11. Goel R, Patel EU, Cushing MM, et al. Association of Perioperative Red Blood Cell Transfusions With Venous Thromboembolism in a North American Registry. *JAMA Surg*. 2018;153(9):826-833. doi:10.1001/jamasurg.2018.1565
12. Chervu NL, Balian J, Verma A, et al. Development of a Surgery-Specific Comorbidity Score for Use in Administrative Data. *Ann Surg*. Published online September 24, 2024. doi:10.1097/SLA.0000000000006544
13. Kreps JM (Secretary), Slater CM (Chief E, Duncan JW (Director). Directive No. 15: Race and ethnic standards for Federal statistics and administrative reporting. 1978.

© 2025 Eyth A et al. *JAMA Network Open*.
