# Supplementary material for: Development and Validation of a Risk Model to Predict Intraoperative Blood Transfusion
Source: JAMA Netw Open. 2025 Apr 17;8(4):e255522. doi: 10.1001/jamanetworkopen.2025.5522 (PMC12006869; doi:10.1001/jamanetworkopen.2025.5522)
Supplement: Supplement 2. — Data Sharing Statement [file jamanetwopen-e255522-s002.pdf]

Data Sharing Statement

Eyth. Development and Validation of a Risk Model to Predict Intraoperative Blood Transfusion. *JAMA Netw Open*. Published April 17, 2025. doi:10.1001/jamanetworkopen.2025.5522

Data
Data available: Yes
Data types: Data (not involving human participants)
How to access data: Data can be made available to researchers upon reasonable request from the corresponding author Matthias Eikermann MD, PhD (14 days response time).
When available: With publication

Supporting Documents
Document types: Statistical/analytic code
How to access documents: Data can be made available to researchers upon reasonable request from the corresponding author Matthias Eikermann MD, PhD (14 days response time).
When available: With publication

Additional Information
Who can access the data: To anyone at reasonable request
Types of analyses: any reasonable purpose
Mechanisms of data availability: Data will be provided by the PI Dr. Eikermann
Any additional restrictions: none
